# Isolation of a complementary bacteriophage from a phage-resistant mutant expands control strategies against *Xanthomonas campestris*

**DOI:** 10.3389/fmicb.2026.1853113

**Published:** 2026-08-03

**Authors:** Qing Yu, Qingshan Wu, Tao Lu, Ruina Yang, Zheng Fang, Lan Xiang, Qiuping Liu, Leitao Tan, Xiaosheng Zhao, Chuangen Lin, Qingbei Weng

**Affiliations:** 1 School of Life Sciences, Guizhou Normal University, Guiyang, China; 2 School of Biological Sciences and Agriculture, Qiannan Normal University for Nationalities, Duyun, China

**Keywords:** bacteriophage, biocontrol, *Caudoviricetes*, phage adsorption, phage cocktail, phage resistance, *Xanthomonas campestris* pv. *campestris*

## Abstract

Bacterial resistance to bacteriophages represents a major limitation for the durable use of phage-based biocontrol strategies in agriculture. Here, we used a phage-resistance derivative of *Xanthomonas campestris* pv. *campestris* (*Xcc*) as a selective host to isolate an additional phage with activity against resistant bacteria. A spontaneous mutant, designated *Xcc* 8004R, was isolated following exposure of *Xcc* 8004 to phage X1. *Xcc* 8004R exhibited a growth profile comparable to that of the wild-type strain, but differed in colony appearance, X1 adsorption efficiency, and motility phenotypes. Whole-genome resequencing identified mutations in genes annotated as encoding a lipopolysaccharide (LPS) core biosynthesis protein, PilY1, N-acetylmuramoyl-L-alanine amidase, and IS1404 transposase, and a conserved hypothetical protein. Using *Xcc* 8004R as the isolation host, we recovered a bacteriophage, vB_Xcc_GYRb1 (GYRb1), from agricultural soil. GYRb1 infected both *Xcc* 8004 and *Xcc* 8004R and exhibited different adsorption kinetics on the two strains. Transmission electron microscopy showed an icosahedral head and a long, non-contractile tail. Genome sequencing revealed a circularly assembled 91,478-bp double-stranded DNA genome encoding 128 predicted open reading frames and two tRNAs. No recognizable antibiotic resistance or bacterial virulence genes were detected. Phylogenomic, ANI, and gene-sharing network analyses suggest that GYRb1 is highly divergent from currently available related phages and may represent a candidate genus-level lineage within the class *Caudoviricetes*. GYRb1 suppressed the growth of *Xcc* 8004 and *Xcc* 8004R *in vitro* and reduced disease severity in cabbage leaf. A cocktail containing GYRb1 and X1 reduced OD_600_-based bacterial regrowth compared with single-phage treatments *in vitro*. Together, these findings support the feasibility of using phage-resistant bacterial mutants as selective hosts to obtain complementary candidate phages, while highlighting the need for receptor identification, resistance-frequency analysis, lysogeny assessment, and broader host-range testing before practical application.

## 1 Introduction

Black rot, caused by *Xanthomonas campestris* pv. *campestris (Xcc),* severely affects *Brassicaceae* production worldwide (Vicente and Holub, 2012) and can cause crop losses of up to 50% and represents a major threat to vegetable production (Rubel et al., 2020). Antibiotics and copper-based compounds have been used to control bacterial plant diseases (Sundin et al., 2016). However, excessive antibiotic use may promote antimicrobial resistance and facilitate horizontal transfer of resistance genes (Sundin and Wang, 2018), whereas copper-based compounds can accumulate in soil and adversely affect plant growth and the surrounding environment (Liu et al., 2020). Therefore, efficient and field-applicable alternatives for black rot management remain urgently needed.

Bacteriophages (phages) have emerged as promising biological agents for controlling plant-associated bacterial pathogens (Hatfull, 2008) and can contribute to plant health (Pratama et al., 2020). As biological agents, phages offer numerous potential advantages due to high host specificity, self-replication ability, lower costs, and no impact on beneficial microbial communities in the environment (Loc-Carrillo and Abedon, 2011). *Xcc-*infecting phages have been isolated from soil (Silva et al., 2019), cabbage leaves (Neoralová et al., 2023), and wastewater (Vique et al., 2025). These phages are primarily classified within the class *Caudoviricetes*, including members of *Carpasinavirus*, *Foxunavirus*, *Foxquatrovirus*, and *Eisenstarkvirus*, in addition to unclassified *Caudoviricetes* (Neoralová et al., 2023), with additional *Xcc* phages reported in the family *Inoviridae* (Lin et al., 2001). Several *Xcc* phages have shown protective effects against black rot. For instance, foliar application of XcpSFC 211 on the leaves reduced the incidence of black rot in broccoli (Nagai et al., 2017), and FoX2 and FoX6 inhibited black rot in cabbage after seed coating, root irrigation, or foliar spraying (Holtappels et al., 2022).

Despite this promise, rapid evolution of bacterial resistance remains a central obstacle to durable phage biocontrol (Li et al., 2022). Phage-resistance mutants can retain pathogenicity *in planta*. For instance, phage-resistant mutants of *Xanthomonas oryzae* pv. *oryzae* (*Xoo*) did not exhibit reduced virulence compared to the wild-type and still caused bacterial leaf blight in rice (Liu et al., 2022). Similarly, phage-resistant mutants of *Pseudomonas syringae* pv. *actinidiae* remained pathogenic on kiwifruit plants (Warring et al., 2022; Tian et al., 2024). Therefore, identifying additional phages that can infect resistant bacterial populations is important for designing more robust phage-based control strategies.

One possible strategy is to exploit phage-resistant mutants as a selective host for isolating complementary phage. Phages that differ in receptor use or infection strategy may complement one another during bacterial control. For example, phages infecting wild-type and mutant strains have been reported to use different receptors, such as outer core oligosaccharide and O-antigen (Kim et al., 2014). Experimental phage evolution has been shown to expand host ranges against antibiotic-resistant isolates (Ghatbale et al., 2025). Directed phage evolution can improve killing activity against specific hosts and extend bacterial suppression (Borin et al., 2021), but outcomes are not always predictable, and evolved phages may lose desirable properties. Meanwhile, rapid anti-phage resistance can emerge in phytopathogenic bacteria under phage pressure, driven by single nucleotide mutations and ongoing phage-host coevolution (Vacheron et al., 2025). Conversely, random screening for phages with diverse infection strategies can be labor-intensive and inefficient (Yang et al., 2020; Li et al., 2022). Using resistant bacteria as selective hosts may provide a practical enrichment strategy for identifying candidate phages that retain activity against specific escape mutants.

In this study, we first isolated and characterized a spontaneous phage X1-resistant mutant of *Xcc* 8004. We then used this mutant as a selective host to isolate a phage, GYRb1, from agricultural soil. Through genomic, phylogenetic, and functional analyses, we show that the newly isolated phage infects both the parental strain and one X1-resistant derivative and reduces bacterial growth and disease severity under laboratory and controlled plant-assay conditions. Our findings provide proof of concept evidence that phage-resistant mutants can be used as selective hosts to expand candidate phage resources for *Xcc* control.

## 2 Materials and methods

### 2.1 Bacterial and phage strains

*Xcc* 8004 (GenBank accession number: CP000050) used in this study is a rifampicin-resistant strain that was spontaneously generated from the cauliflower isolate *Xcc* NCPPB No. 1145 (Qian et al., 2005). *Xcc* 8004 was stored in 25% (v/v) glycerol solution at −80 °C in the laboratory. It was cultivated with nutrient yeast glycerol (NYG) broth (5.0 g peptone, 3.0 g yeast extract, and 20.0 g glycerol, 1 L distilled water) at 28 °C and 180 rpm. Phage X1 was previously isolated from cave sediment using *Xcc* 8004 as the host bacterium (Wu et al., 2026). The phage (10^8^ plaque-forming units (PFU)/mL) was mixed with the host cells in the exponential phase (1.25 × 10^8^ colony-forming units (CFU)/mL, OD_600_ = 0.6) and incubated at 28 °C with shaking at 180 rpm for 4 h. After centrifugation at 12,000 × g for 20 min at 4 °C, the supernatant was then filtered through a 0.22 μm filter membrane. The phage supernatant was stored in sodium chloride-magnesium sulfate (SM) buffer (100 mM NaCl, 8 mM MgSO_4_, 5 mM Tris–HCl (pH 7.5), 0.01% gelatin) at 4 °C, or was mixed with sterile glycerol at a final concentration of 25% and stored at −80 °C for long periods of time. The titer was determined by double-layer agar method.

### 2.2 Isolation of phage-resistant mutant

After phage X1 was co-cultured with *Xcc* 8004 at a multiplicity of infection (MOI) of 0.01 for 24 h at 28 °C, the resistant clones were isolated by streak plating on NYG plate (1.8% agar, w/v) and then screened by phage spot assay to confirm their resistant to phage X1. Briefly, the colonies were inoculated into 5 mL NYG broth and cultivated at 28 °C and 180 rpm overnight, respectively, 1 mL of the culture was mixed with 10 mL NYG semi-solid medium (0.75% agar, w/v) and overlaid onto the NYG solid plate. Then, phage X1 lysate (10^8^ PFU/mL) was 10-fold serially diluted with SM buffer, and 5 μL of each dilution was spotted onto the lawn and then incubated at 28 °C for 12 h. A clone that persistently showed no plaque after three successive streak plating was selected as the resistant mutant and designated *Xcc* 8004R. The resistance of *Xcc* 8004R to the phage X1 remained stable throughout multiple passages.

*Xcc* 8004 and *Xcc* 8004R (OD_600_ = 0.3) were inoculated at 2% (v/v) into 30 mL of NYG liquid medium and incubated at 28 °C with 180 rpm shaking. Samples were collected every 2 h over a 24 h period to measure OD_600_ values and bacterial colony counts were determined. Growth curves were plotted with time on the X-axis, and OD_600_ values and colony counts were displayed on separate Y-axis. All experiments were performed in triplicate.

Phage X1 lysate was mixed with bacteria culture at an MOI of 0.1, and incubated at 28 °C at 180 rpm for 30 min. The supernatant was collected after centrifugation at 12,000 × g, and then passed through a 0.22 μm filter. The phage titers remaining in the supernatant were evaluated based on the assay of double layer plates with *Xcc* 8004. Adsorption = (1 − titers of free phages / titers of initial phages) × 100%.

To test the swimming motility of the strains, bacteria were transferred with a sterile toothpick and stabbed into 0.28% agar plate containing 0.03% bacto peptone and 0.03% yeast extract. The plate was then incubated at 28 °C for 3 days. To analyze the characteristics of the swarming motility, the overnight-cultured bacteria (NYG medium, OD_600_ = 0.6) were inoculated onto a NY plate containing 2% glucose and 0.6% agar, and then incubated at 28 °C for 3 days (Qi et al., 2020).

Bacterial genomic DNA was extracted using Tianamp Bacteria DNA Kit (Tiangen biochemical technology (Beijing) Co. Ltd.) and the DNA concentration and purity were determined at 260 nm and 280 nm using Nanodrop spectrophotometry. Sequencing was performed by Personabio Biotech (Personabio, Shanghai, China) using Illumina NovaSeq PE150 platform. The sequencing reads were aligned to the reference genome using the BWA-MEM (0.7.12-r1039) program (Li and Durbin, 2009). Genomic alignment was conducted between *Xcc* 8004R and the reference genome *Xcc* 8004. Single nucleotide polymorphism (SNP) and insertion and deletion (indel) variants were identified using the MUMmer tools (Chiaromonte et al., 2002). The variant sites were annotated using the ANNOVAR software (Wang et al., 2010).

### 2.3 Phage isolation, purification, and propagation

Phage was isolated from the surface soil collected from Chinese cabbage (*Brassica rapa* sp. *pekinensis*) field in Guiyang, Guizhou Province, China (26°26′N, 106°40′E). Phages were isolated and purified using double-layer agar method. Specifically, 10 g of the soil sample and 1 mL of overnight *Xcc* 8004R culture were added to 40 mL of NYG broth. After incubation overnight at 28 °C and 180 rpm, the suspension was centrifuged for 20 min, 12,000 × g at 4 °C and then filtered through a 0.22 μm membrane. Then 1 mL of the filtrate and 1 mL *Xcc* 8004R culture were added to 10 mL NYG semi-solid medium, and overlaid onto a NYG solid plate. After incubation overnight at 28 °C, the plaques were isolated and purified, designated vB_Xcc_GYRb1 (GYRb1 for short).

### 2.4 Transmission electron microscopy

Purified phage suspension (10^8^ PFU/mL) was adsorbed onto carbon-coated copper grids and then negatively stained with 2% phosphotungstic acid. After drying at room temperature, phages were subsequently observed using transmission electron microscopy (TEM) (HT7800, HITACHI, Japan) at an accelerating voltage of 200 kV (Chen et al., 2020).

### 2.5 Determination of phage host range

The host range of GYRb1 was initially determined using spot assays, and productive infection of susceptible strains was further quantified by measuring the efficiency of plating (EOP). Spot-test clearing was not interpreted as evidence of productive infection unless confirmed by plaque formation and EOP determination. A panel of six bacterial strains was selected for host susceptibility testing. Among these, *Xcc* ATCC 33913 and *X. campestris* pv. *badrii* ATCC 11672 were obtained from Beijing Boyihuike Biotechnology Co., Ltd. (China). *Xcc* XC1 was a kind gift from Professor Kaihuai Li (School of Life Sciences, Guizhou University). *X. oryzae* pv. *oryzae* 2212 was provided by Professor Meiyan Yang (College of Food Science, South China Agricultural University). For the spot assay, each strain was cultured in 5 mL of NYG medium at 28 °C with shaking until the OD_600_ reached 0.6. The cultures were then centrifuged at 5,000 × g for 10 min and resuspended in 5 mL of SM buffer. Subsequently, 0.1 mL of the bacterial suspension was mixed with 4.5 mL of molten soft agar and overlaid onto NYG agar plates. After solidification, 10 μL of phage suspension (10^7^ PFU/mL) was spotted onto the plates, which were then incubated at 28 °C for 12 h. For EOP determination, the phage titer obtained on each test strain was compared with that obtained on the reference host *Xcc* 8004R. EOP values were categorized according to previously established criteria as high (EOP ≥ 0.5), moderate (0.1 ≤ EOP < 0.5), low (0.001 ≤ EOP < 0.1), or undetectable (Wu et al., 2026).

### 2.6 Stability determination of phage

To determine phage sensitivity to chloroform, the phage lysate (10^7^ PFU/mL) was mixed with chloroform with 25% (v/v) final concentration and left at room temperature for 10 min. After centrifugation at 10,000 × g for 3 min, the phage titer of the supernatant was measured. For the temperature stability test, 1 mL of phage lysate (10^7^ PFU/mL) was incubated at 4 °C, 20 °C, 30 °C, 40 °C, 50 °C, 60 °C, and 70 °C for 1 h, respectively. The titers were then measured. For the pH stability test, 100 μL of phage lysate (10^7^ PFU/mL) was added to 900 μL of SM buffer with pH values of 3, 4, 5, 6, 7, 8, 9, 10, 11, and 12 (adjusted with 1 mol/L HCl or 1 mol/L NaOH). The phage titers were measured after incubation for 1 h at 28 °C for 1 h. The phage lysate (10^7^ PFU/mL) was exposed to ultraviolet (UV) light (30 W, 50 cm) for 0 min, 20 min, 40 min, 60 min, 80 min, 100 min, and 120 min. The phage titers were determined. All experiments were performed in triplicate and three technical replicates.

### 2.7 Optimal MOI and one-step growth curve

The phage was mixed with bacterial culture in proportion (100, 10, 1, 0.1, 0.01, and 0.001), and incubated at 28 °C for 6 h. The phage titers were determined, and the ratio producing the highest phage titer was considered the optimal MOI (Li et al., 2019). The experiments were performed in triplicate for each proportion.

The one-step growth curve was performed. Briefly, the phage was mixed with bacteria at an MOI of 0.01 and incubated at 28 °C for 15 min for adsorption. After centrifugation at 10,000 × g for 1 min at room temperature, the pellets were resuspended in 10 mL of NYG broth and incubated with shaking (180 rpm) at 28 °C. During the 6 h incubation, 500 μL of the sample was collected at 30-min intervals to determine the phage titer. The experiment was performed with three biological replicates and three technical replicates.

### 2.8 Adsorption assay

Phage lysate was mixed with bacteria culture at an MOI of 0.1, and incubated at 28 °C at 180 rpm. A total of 500 μL of the mixture were collected every 5 min for up to 30 min. The supernatant was collected after centrifugation at 12000 × g, and then passed through a 0.22 μm filter. The phage titers at each time point were determined (Chen et al., 2023). All experiments were performed with three biological replicates and three technical replicates.

### 2.9 *In vitro* bacterial killing assay

Fresh bacteria culture (OD_600_ = 0.1) was mixed with phage in a 96-well microplate at final MOIs of 0.001, 0.01, 0.1, 1, 10, and 100, respectively. Subsequently, the mixture was incubated at 28 °C under shaking at 180 rpm, then the OD_600_ was measured at 2 h intervals for up to 36 h. NYG broth was used instead of the phage suspension for blank control. The experiments were performed with three biological replicates and three technical replicates.

### 2.10 Phage DNA extraction, genome sequencing and annotation

The phage genomic DNA was extracted using the TaKaRa MiniBEST Viral RNA/DNA Extraction Kit, and the DNA concentration and purity were determined at 260 nm and 280 nm using Nanodrop spectrophotometry. Sequencing was performed by Magigene (Guangdong Biotechnology Co., Ltd., China) using an Illumina NovaSeq platform. The obtained FastQ raw reads were processed with SOAPnuke v2.0.5 (Chen et al., 2018) for adapter trimming, quality-based trimming, and short reads were filtered. *De novo* assembly was performed using MEGAHIT v1.1.2 (Li et al., 2016). CheckV v1.0 was used to evaluate the results for obtaining the final genome sequence (Nayfach et al., 2021). Genome circularity was assessed by identifying terminal overlap in the assembled contig and by remapping quality-filtered reads to the circularized assembly to confirm continuous read coverage across the junction.

The open reading frames (ORFs) of the genome sequence were predicted using GeneMarkS^1^ and RAST server.^2^ The functional annotation of ORFs was performed by BLASTp^3^ analysis against the non-redundant protein database. Additionally, tRNA genes were identified with tRNAscan-SE^4^ (Chan and Lowe, 2019) using default parameters. Conserved domains in the ORFs were identified using the CD-Search server.^5^ The phage GYRb1 genome map was generated using Proksee^6^ based on gene annotations. Antimicrobial resistance genes (ARGs) and virulence factors were screened against the Comprehensive Antibiotic Resistance Database (CARD, https://card.mcmaster.ca/analyze/rgi) (Alcock et al., 2023) and Virulence Factor Database (VFDB, http://www.mgc.ac.cn/VFs/) (Liu et al., 2019). The lifestyle of phages is predicted using the PhageAI tool.^7^

### 2.11 Phylogenetic analysis and comparative genomic analysis

The whole genome sequence of phage GYRb1 was aligned against the GenBank database (accessed on 10 August 2025) using BLASTn and the top 16 related phage genomes were retained. Additionally, BLASTp analysis of the phage terminase large subunit (TerL) amino acid sequence identified 3 similarity sequences. After removing repetitive sequences and metagenome-assembled genomes (MAGs)-derived sequences, a combined dataset including 14 phage genomes including GYRb1 was obtained for subsequent analysis. Viptree^8^ (Nishimura et al., 2017) was used to generate a proteomic tree. TerL phylogenic tree was constructed by Maximum Likelihood (ML) method using MEGA (v11.0.13) under the WAG matrix model and 1,000 bootstrap replicates.

Genomic network analysis of phage GYRb1 was conducted using vConTACT (v2.0) (Bin Jang et al., 2019) and the INfrastructure for a PHAge REference Database (INPHARED) (https://millardlab.org/phage-genome-jan2025/, accessed February 2025) (Cook et al., 2021). Based on the number of shared protein clusters (PCs) between genomes, closely related genomes were grouped into virus clusters (VCs) using the Markov cluster (MCL) and clustering with overlapping neighborhood expansion (ClusterONE) (v1.0). The resulting network was visualized using Cytoscape (v3.7.2) software (Shannon et al., 2003).

The whole genome sequence of phage GYRb1 was compared with that of its closest relative using Easyfig software (Kumar et al., 2018). Genome-wide average nucleotide identity (ANI) values for phage GYRb1 and the related phages were calculated and visualized as a heatmap using the Virus Intergenomic Distance Calculator (VIRIDIC)^9^ (Moraru et al., 2020).

### 2.12 *In vitro* bacterial killing assay of phage

Phage and the host culture were mixed (MOI = 0.01) individually and plated on the double-layer agar plate. Phage X1 was proliferated using *Xcc* 8004 as the host, whereas phage GYRb1 was proliferated with *Xcc* 8004R. After overnight incubation at 28 °C, plates showing complete lysis were harvested by suspending the top agar layer in 10 mM MgCl_2_ solution. The suspension was centrifuged at 12000 × g for 20 min at 4 °C, and then filtered through a 0.22 μm membrane. Phage titers were determined and adjusted to 10^6^ PFU/mL with 10 mM MgCl_2_ solution. Subsequently, GYRb1 was mixed with X1 at a 1:1 ratio (v/v) for subsequent experiments.

*Xcc* 8004 culture (OD_600_ = 0.1) and the phage suspension were mixed (MOI = 0.01) and 100 μL of the mixture was added in a 96-well microplate. The MgCl_2_ solution was used instead of the phage was used as a control. The plate was incubated at 28 °C with 180 rpm shaking and the OD_600_ was measured every 2 h for 24 h. Experiments were performed in triplicate.

### 2.13 *In vivo* inhibition of bacteria assay

The seeds of cabbage Jingfeng No.1 (*Brassica oleracea* var. *capitata*) were sown in seedling trays. After 2 weeks of germination, seedlings were transplanted into pots (13 × 12 cm) and cultivated under natural light condition at 25–30 °C with 60–70% relative humidity. Plants were treated with normal water supply throughout the growth period. Uniformly sized plants were selected at the four- to five-leaf stage for the prophylactic protection assay.

The prophylactic protection efficacy of phage GYRb1 against *Xcc* 8004 and *Xcc* 8004R *in vivo* was evaluated by foliar spraying (FS) (Wu et al., 2026). Four size-matched, fully expanded leaves per plant were selected and sprayed with 1 mL of phage suspension (10^6^ PFU/mL) using a spray bottle. After 24 h, the overnight bacterial culture (adjusted to OD_600_ = 0.1 with 10 mM MgCl_2_) was inoculated using a sterile-clip method. Sterile scissors were dipped in the bacterial culture and used to make perpendicular incisions 1–2 cm from leaf tips, ensuring that the wound was in full contact with the bacterial culture. The experiment included three biological replicates with six cabbage plants used for each replicate. Controls included three groups: (1) positive control (leaves inoculated with bacteria without phage pretreatment), (2) negative control (leaves sprayed with phage but not inoculated with bacteria), and (3) blank control (10 mM MgCl_2_ solution instead of bacterial culture). The cabbage plants were maintained in a light incubator under controlled conditions at 28 °C with 90% humidity with a photoperiod of 16 h. After 14 days, the pathological symptoms were documented and the disease severity was quantified by measuring the length (cm) of V-shaped chlorotic lesions along leaf veins using digital calipers.

### 2.14 Statistical analysis

Statistical analyses were performed using GraphPad Prism v9.3. For comparisons between two groups, an unpaired two-tailed Student’s *t*-test was used when data met normality and variance-homogeneity assumptions. For experiments involving multiple treatments at a single time point, one-way analysis of variance (ANOVA) followed by Tukey’s multiple-comparison test was used. For time-course experiments, two-way ANOVA with treatment and time as factors was used, followed by appropriate multiple-comparison correction. All experiments were performed in triplicate, biological replicates were treated as the unit of statistical analysis. The results data are expressed as mean ± standard deviation (SD). A *p* value <0.05 was considered statistically significant.

## 3 Results

### 3.1 Isolation of a spontaneous phage resistant mutant *Xcc* 8004R

**Figure 1:**
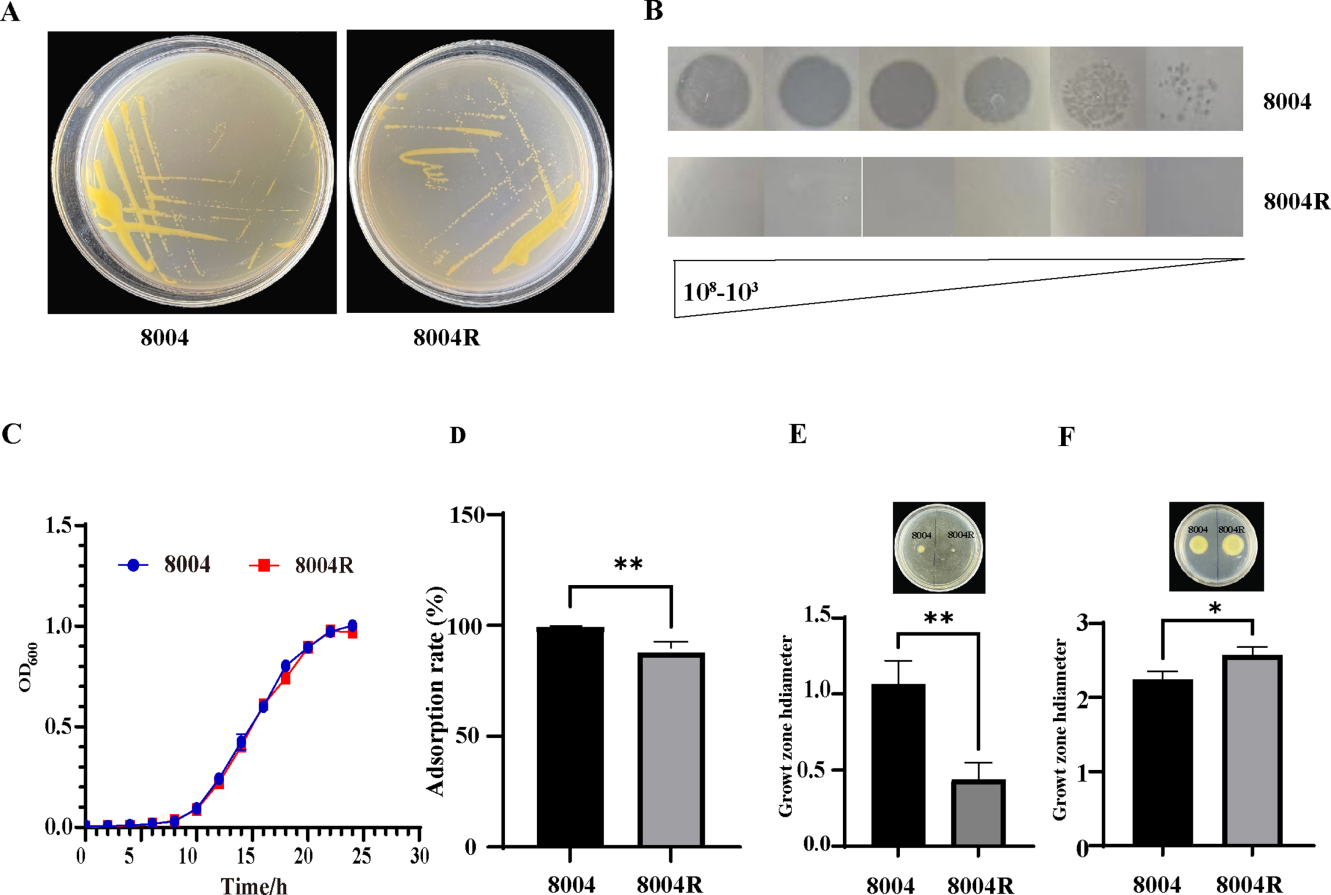
Identification and characterization of phage-resistant strain *Xcc* 8004R. **(A)** Colony morphology of *Xcc* 8004 and *Xcc* 8004R. **(B)** Spot assay demonstrating the resistance of *Xcc* 8004R to phage X1. Tenfold serial dilutions of phage X1 (ranging from 10^8^ PFU/mL to 103 PFU/mL) were spotted onto bacterial lawns of *Xcc* 8004 and *Xcc* 8004R. **(C)** Growth curves of *Xcc* 8004 and *Xcc* 8004R. **(D)** The adsorption efficiencies of phage X1 toward *Xcc* 8004 and *Xcc* 8004R. **(E)** Swimming motility. Swimming motility of *Xcc* 8004 and *Xcc* 8004R was assessed on semi-solid agar plates (0.28% agar) after 3 days of incubation at 28 °C. The diameter of the motility zone was measured to evaluate bacterial movement. **(F)** Swarming motility. Swarming motility was assessed by inoculating overnight-cultured bacteria onto NY plates containing 2% glucose and 0.6% agar. Plates were incubated at 28 °C for 3 days. The migration zone diameter was recorded to evaluate swarming ability. Data represent the mean ± standard deviation (*SD*) of three independent replicates. Error bars indicate *SD*. ‘Statistically significant differences among the treatments are indicated by * (*p* < 0.05) and ** (*p* < 0.01)’.

Following co-cultivation of phage X1 with its susceptible host *Xcc* 8004, a mutant strain exhibiting resistance to X1 under the spot assay conditions was isolated and designated *Xcc* 8004R. On NYG solid medium, *Xcc* 8004R colonies exhibited a smooth, moist, and opaque, appearing lighter in color compared to the wild-type *Xcc* 8004 (Figure 1A). A spot assay confirmed the resistance of *Xcc* 8004R to phage X1, as no plaques were observed on the *Xcc* 8004R lawn (Figure 1B). The growth dynamics indicated that the growth rate of *Xcc* 8004 and *Xcc* 8004R was comparable (Figure 1C). The adsorption efficiency of phage X1 to *Xcc* 8004 (99.17%) was significantly higher than that to *Xcc* 8004R (87.64%) (*p* < 0.01) (Figure 1D). Additionally, the swimming motility of *Xcc* 8004R was significantly weaker than that of *Xcc* 8004 (*p* < 0.01) (Figure 1E), but its swarming motility was significantly stronger than that of *Xcc* 8004 (*p* < 0.05) (Figure 1F).

To identify genomic changes potentially associated with the resistant phenotype, whole-genome resequencing of *Xcc* 8004R identified 191 sequence differences relative to the *Xcc* 8004 reference genome. Seven variants were located in coding regions or selected for detailed annotation (Table 1), including variants in genes annotated as coding lipopolysaccharide (LPS) core biosynthesis proteins, PilY1, N-acetylmuramyl-L-alanine amidase, IS1404 transposase, and conserved putative proteins (Supplementary Figures S1–S5). Because these variants were not genetically reconstructed or complemented, they should be interpreted as candidate resistance-associated mutations rather than confirmed determinants of X1 resistance.

**Table 1:** The mutation sites in the genomic sequence of phage-resistant mutant *Xcc* 8004R.

Scaffold	Position	Type	Nucleotide substitution	Amino acid substitution	Annotation
scaffold4	234,232	SNP	T → C	S5G	N-acetylmuramoyl-L-alanine amidase
	235,234	SNP	A → G	–	
scaffold14	68,934	SNP	A → G	D106G	lipopolysaccharide core biosynthesis protein
	69,404	SNP	T → C		
scaffold89	139	SNP	T → C	V194A	IS1404 transposase
scaffold3	269,580	SNP	A → G	–	conserved hypothetical protein
scaffold16	17,939	InDel	T →	T481*	PilY1 protein

* indicates a premature stop codon caused by the mutation, resulting in protein truncation with no corresponding amino acid.

**Figure 2:**
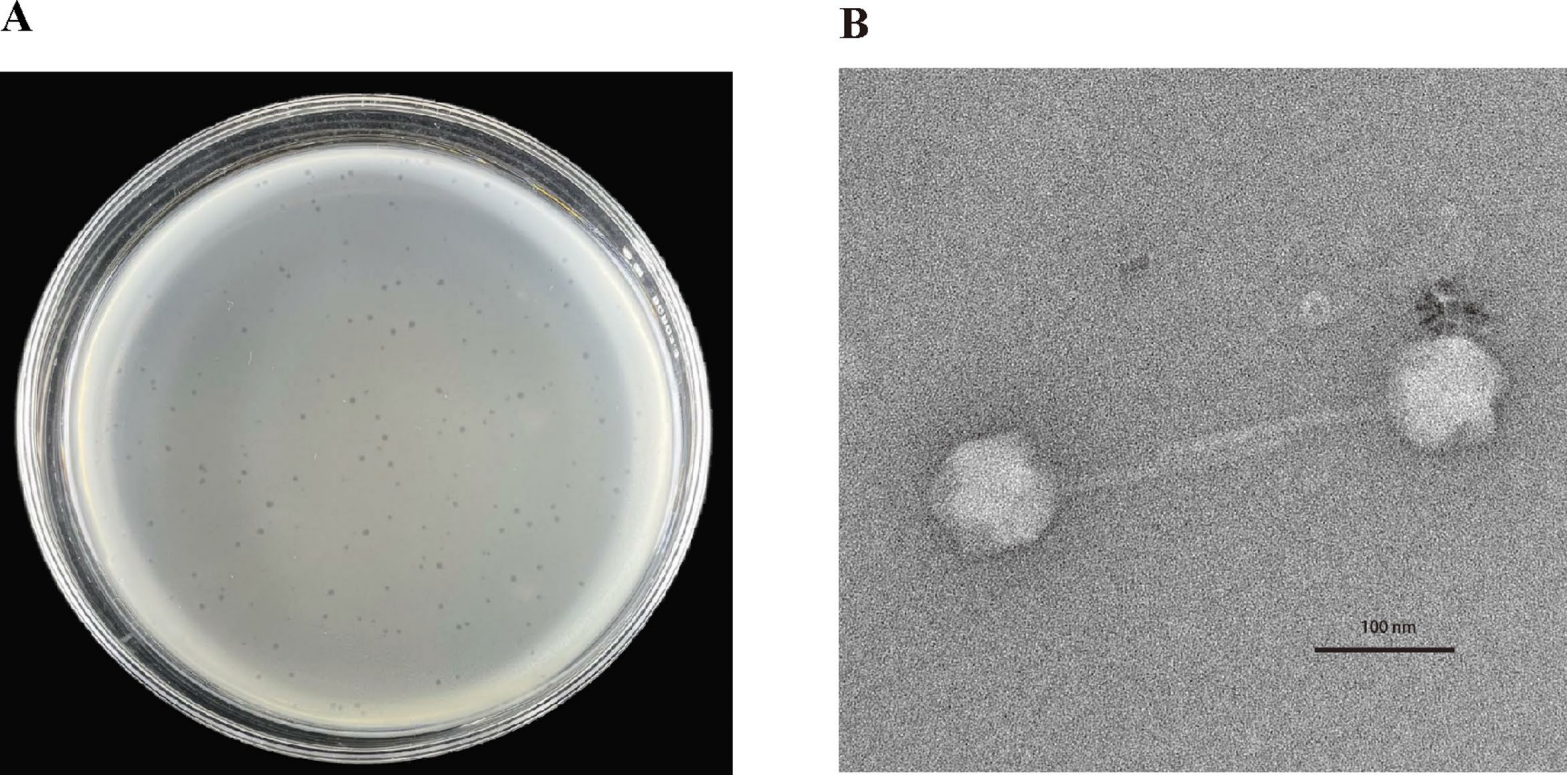
Morphology of phage GYRb1. **(A)** Plaque morphology of GYRb1 on the *Xcc* 8004R lawn. **(B)** Transmission electron micrograph of phage GYRb1. Scale bar: 100 nm.

To isolate a phage capable of infecting this resistant mutant, screening was conducted using *Xcc* 8004R as the host. A novel phage, designated GYRb1, was subsequently isolated from the cabbage field soil collected in Guiyang. This phage formed clear plaques with a diameter of 0.60 ± 0.16 mm on the *Xcc* 8004R lawn (Figure 2A). TEM analysis revealed that phage GYRb1 possesses an icosahedral head (diameter: 89.2 ± 2.0 nm) and a long, non-contractile tail (length: 166.8 ± 3.0 nm) (Figure 2B).

### 3.2 Host range and stability of phage GYRb1

Spot assays indicated that GYRb1 could lyse *Xcc* 8004, *Xcc* 8004R, *Xcc* ATCC 33913, and *X. oryzae* pv. *oryzae* 2,212, whereas no lytic activity was detected against *Xcc* XC1 and *X. campestris* pv. *badrii* ATCC 11672. EOP assays further confirmed productive infection on susceptible strains, with *Xcc* 8004 showing high sensitivity (EOP > 0.5), whereas *Xcc* ATCC 33913 and *X. oryzae* pv. *oryzae* 2,212 exhibited low sensitivity (0.001 < EOP < 0.1) (Table 2).

**Table 2:** Host range and stability of phage GYRb1.

Bacterial strain	Sensitivity to GYRb1	Source	EOP
*Xcc* 8004R	+	Separate from this study	1
*Xcc* 8004	+	*Brassica oleracea var. botrytis*	0.68 ± 0.13
*Xcc* XC1	−	*Brassica oleracea*	−
*Xcc* ATCC 33913	+	*Brassica oleracea var. gemmifera*	0.0021 ± 0.0003
*X. oryzae* pv. *oryzae* 2212	+	*Oryza sativa*	0.0014 ± 0.0002
*X. campestris* pv. *badrii* ATCC 11672	−	*Xanthium strumarium*	−

+: indicates that the phage can infect the strain, −: indicates that the phage cannot infect the strain.

**Figure 3:**
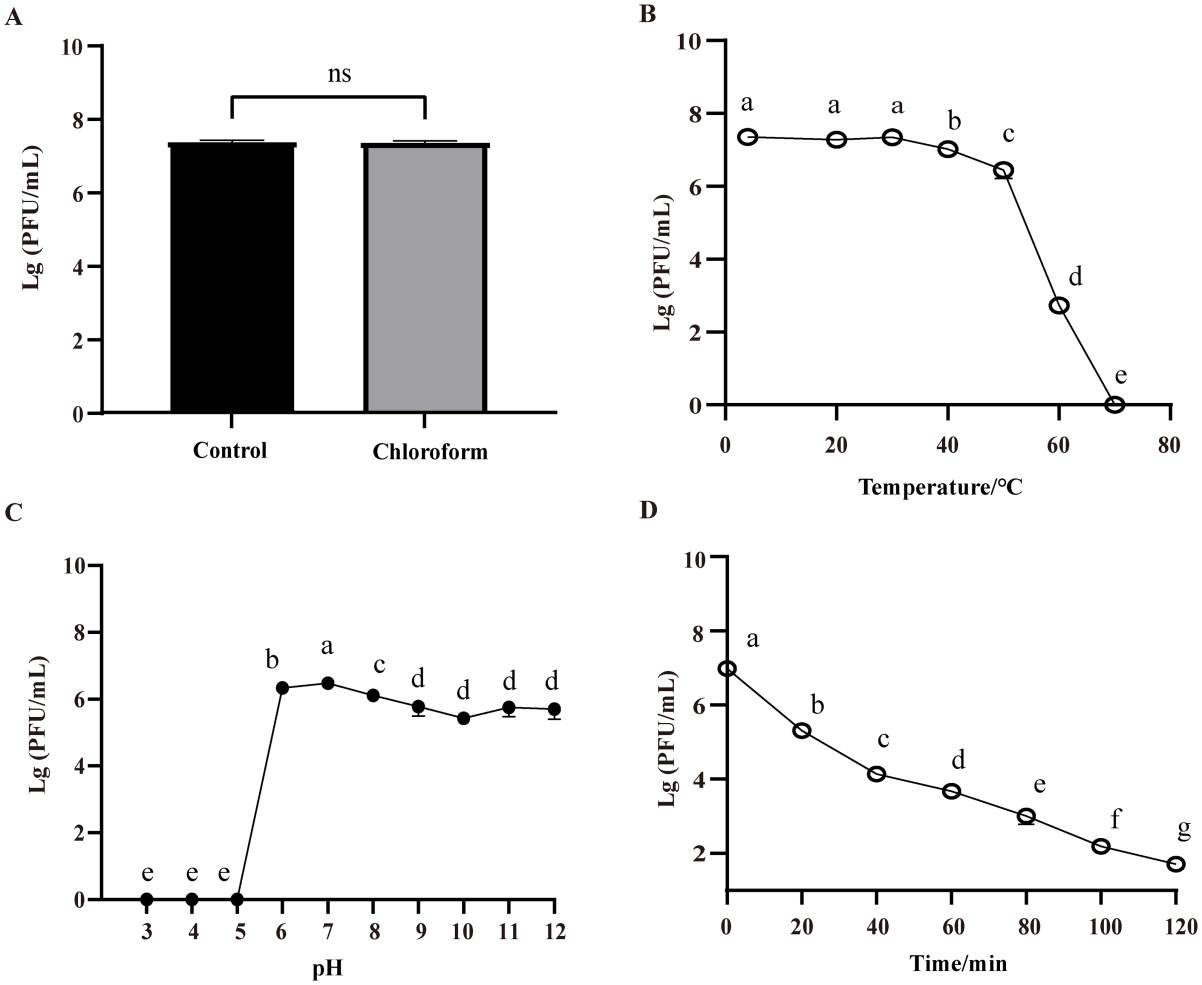
Stability profiles of phage GYRb1. **(A)** Chloroform stability. Phage (10^7^ PFU/mL) was treated with 25% chloroform for 10 min at room temperature, and residual titer was measured after centrifugation. **(B)** Thermal stability. Phage (10^7^ PFU/mL) was incubated at 4 to 70 °C for 1 h, and residual titers were determined. **(C)** pH stability. Phage (10^7^ PFU/mL) was incubated in SM buffer at pH 3 to 12 for 1 h at 28 °C, and residual titers were measured. **(D)** UV stability. Phage (10^7^ PFU/mL) was exposed to UV light (30 W, 50 cm) for 0 to 120 min, and residual titers were determined. Data represent the mean ± standard deviation (SD) of three independent replicates. Error bars indicate SD. Means labeled with different letters are significantly different (*p* < 0.05); ns: Not significant (*p* > 0.05).

The stability of phage GYRb1 against chloroform, temperature, pH, and UV irradiation were evaluated. The phage titer remained unchanged after treatment with chloroform (25% v/v), indicating chloroform tolerance (Figure 3A, *p* > 0.05). Thermal stability assays showed that the phage was stable between 4 °C to 40 °C; however, the titer decreased markedly at 50 °C (*p* < 0.05) and was completely abolished at 70 °C (Figure 3B). The phage retained high activity (titer ranging from 2.69 × 10^5^ to 2.17 × 10^6^ PFU/mL) with a pH range of 6 to 12 but completely inactivated at pH values below 5 (Figure 3C). UV irradiation led to a time-dependent reduction in phage titer. After 20 min of exposure, the titer decreased significantly from 9.63 × 10^6^ PFU/mL to 1.37 × 10^4^ PFU/mL (*p* < 0.01), declining further to 51 PFU/ mL after 120 min (Figure 3D).

### 3.3 Optimal MOI and one-step growth curve of phage GYRb1

**Figure 4:**
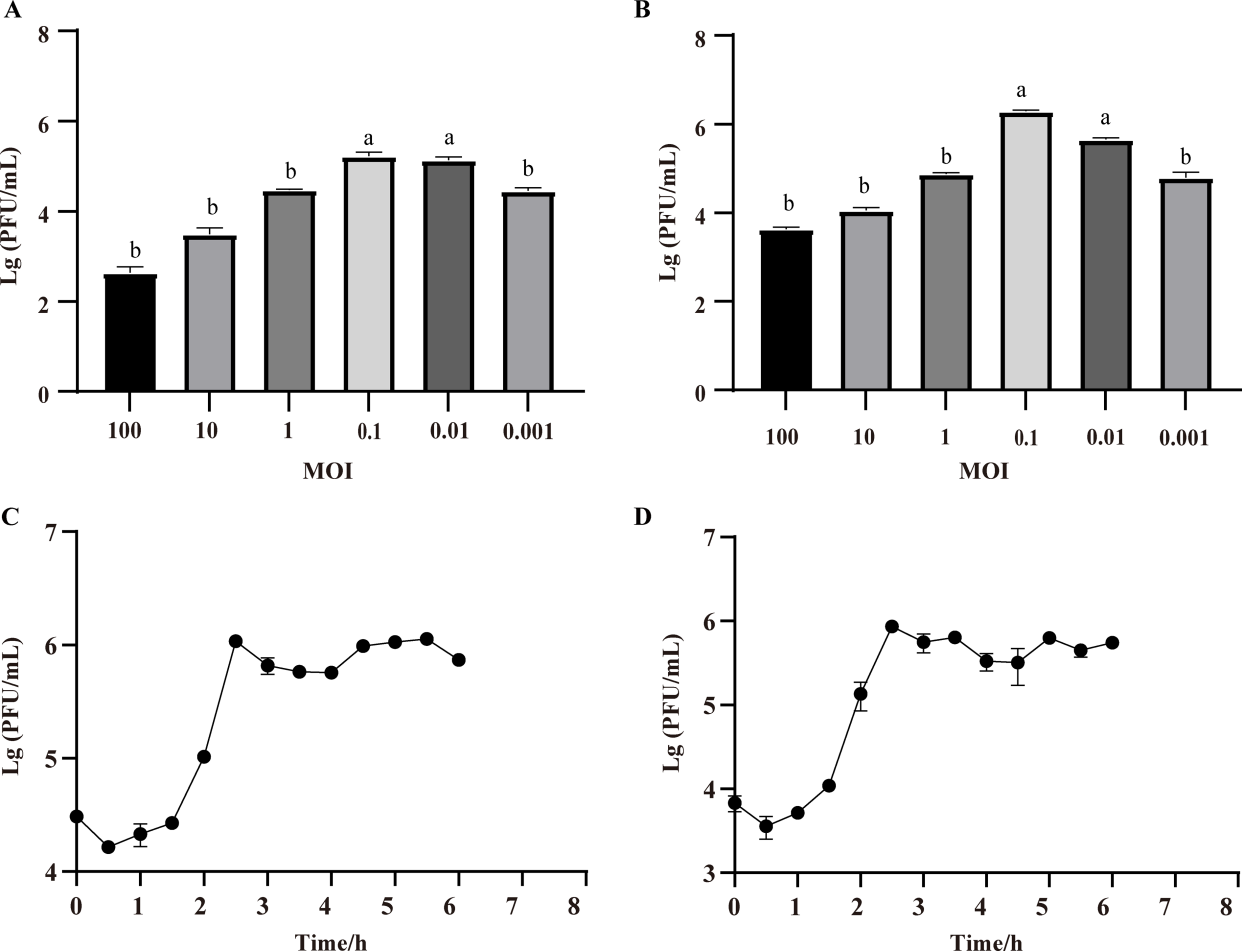
Optimal MOI and one-step growth curve of phage GYRb1. Optimal MOI for infection of **(A)** *Xcc* 8004 and **(B)** *Xcc* 8004R. Phage GYRb1 was mixed with *Xcc* 8004 at different MOIs (0.001, 0.01, 0.1, 1, 10, and 100) and incubated at 28 °C for 6 h. The MOI producing the highest phage titer was determined as optimal. One-step growth curves of GYRb1 in **(C)** *Xcc* 8004 and **(D)** *Xcc* 8004R. Phage was mixed with bacteria at MOI = 0.01 at 28 °C for 15 min. After centrifugation, the pellet was resuspended in NYG broth and shaken at 28 °C. Samples were collected every 30 min over 6 h for titer determination. Data are presented as mean ± SD of three replicates.

The highest final titers were obtained at MOIs of 0.1 and 0.01 in *Xcc* 8004 and *Xcc* 8004R cultures. Notably, the final phage titer appeared higher in *Xcc* 8004R (1.95 × 10^6^ PFU/mL) (Figure 4B) than in *Xcc* 8004 (1.69 × 10^5^ PFU/mL, *p* < 0.05) (Figure 4A). One-step growth curve analysis performed at an MOI of 0.01 revealed that in *Xcc* 8004, phage GYRb1 exhibited a latent period of 1.5 h, a rise period from 1.5 to 2.5 h, and a burst size of 27 PFU/cell (Figure 4C). In contrast, infection of *Xcc* 8004R resulted in a latent period of 1 h, a rise period from 1 to 2.5 h, and a burst size of 80 PFU/cell (Figure 4D).

### 3.4 *In vitro* bacteriolytic activity of phage GYRb1 on *Xcc* 8004 and *Xcc* 8004R

**Figure 5:**
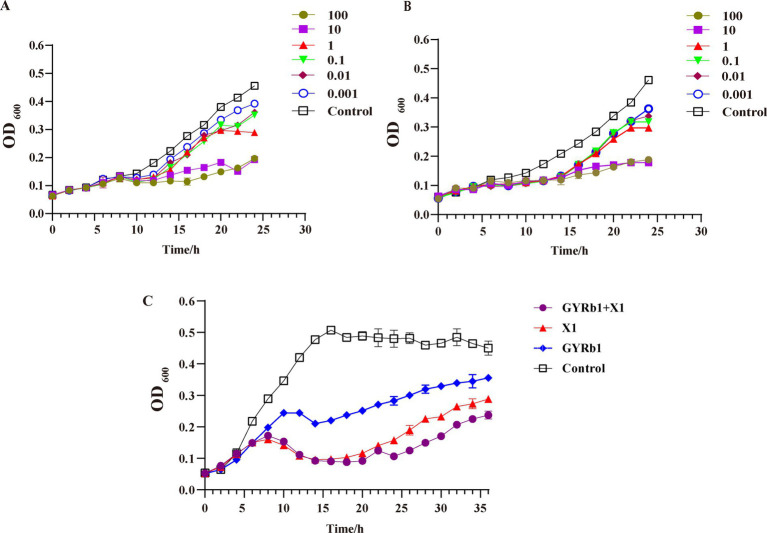
Inhibitory effect of phage GYRb1 on *Xcc* 8004 and *Xcc* 8004R. Growth curves of *Xcc* 8004 **(A)** and *Xcc* 8004R **(B)** in the presence of GYRb1 at varying MOI (100, 10, 1, 0.1, 0.01, and 0.001). **(C)** Inhibitory effect of phage cocktail. Growth curves of *Xcc* 8004 treated with phage cocktail (GYRb1 + X1, 1:1, MOI = 0.01), X1, and GYRb1. OD_600_ was measured every 2 h for 36 h. The bacteria cultures without phage infection were used as the controls. Data are presented as mean ± SD of three independent replicates. Error bars represent SD.

The inhibitory effect of phage GYRb1 against bacterial growth was evaluated by monitoring the growth kinetics of *Xcc* 8004 and *Xcc* 8004R. The results indicated that GYRb1 suppressed the growth of both strains. At higher MOI (100 and 10), the phage exerted a more prolonged inhibitory effect on the bacterial populations (Figures 5A,B).

The antibacterial activity of the phage cocktail (GYRb1 + X1, 1:1, MOI = 0.01) against *Xcc* 8004 was comparable to that of X1 alone. Within 8 h after treatment of *Xcc* 8004, the OD_600_ of GYRb1-treated culture decreased rapidly (*p* < 0.01), but the inhibitory effect of GYRb1 was weaker than that of X1 (*p* < 0.01). Bacterial regrowth was observed during single-phage treatment with either GYRb1 or X1, as reflected by a slight increase in OD_600_ between 15 h-20 h after infection. The OD_600_ values of the phage cocktail at 24 h after infection were lower than those of X1 alone (*p* < 0.05) and persisted through by 36 h monitoring period, indicating that the cocktail reduced bacterial regrowth under the tested conditions (Figure 5C).

### 3.5 Genomic characteristics of GYRb1

Genome sequencing revealed that GYRb1 possesses a circularly assembled double-stranded DNA (dsDNA) genome of 91,478 bp with a GC content of 66.55% (GenBank accession number: PQ569993). Annotation results indicated that the genome contains 128 open reading frames (ORFs) and 2 tRNAs, with 103 ORFs located on the positive strand and 25 ORFs on the negative strand. Among these ORFs, the majority initiate with the start codon ATG (79.55%), 23 ORFs start with GTG (17.56%), and 3 ORFs start with TTG (2.29%). The total length of the ORFs is 87,616 bp, accounting for 95.78% of the whole genome.

**Figure 6:**
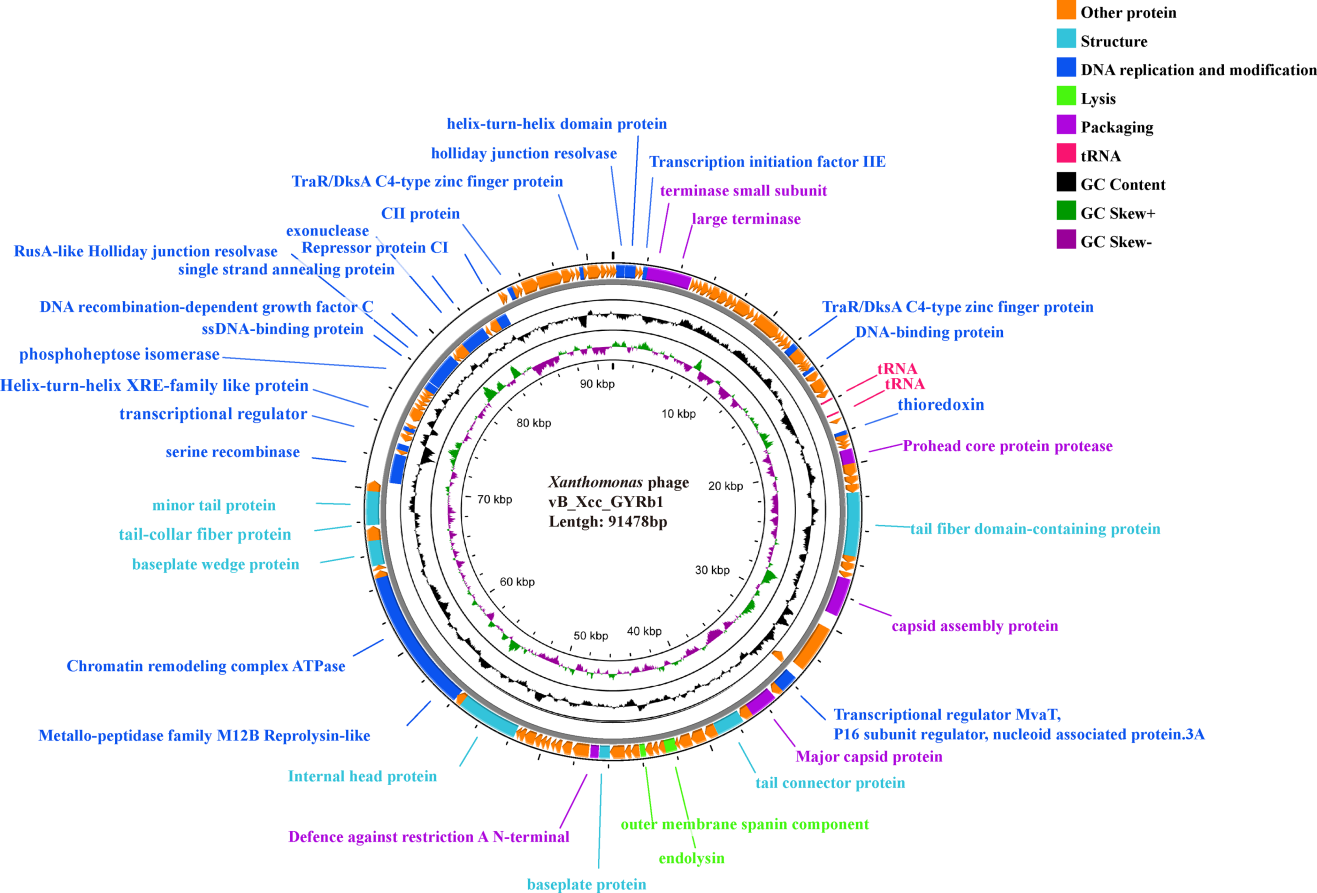
Genome map of phage GYRb1. The complete genome of phage GYRb1 was sequenced using the Illumina NovaSeq platform (paired-end mode). ORFs were predicted using GeneMark and RAST, and annotated by BLASTp against the NCBI nr database and conserved domain database. tRNA genes were predicted using tRNAScan-SE. The genome map was constructed and visualized using Proksee. The outermost circle represents ORFs on the positive strand, the second circle shows ORFs on the negative strand, and different colors represent different gene functions. The third circle displays the GC skew, and the fourth circle indicates the GC content.

Functional annotation of the ORFs was performed via BLASTp search against non-redundant protein database. Of the 128 ORFs, 36 were predicted to encode proteins with known functions, 56 encode hypothetical proteins, while 36 showed no sequence similarity to any annotated proteins in the databases. Based on their predicted functions, the ORFs were categorized into four core functional modules: 7 encoding structural proteins, 6 involved in DNA packaging, 21 associated with DNA replication/modification, and 2 implicated in phage lysis (Supplementary Table S1). CI and CII-like regulatory proteins were predicted in the GYRb1 genome, although the integrase was not predicted. Through PhageAI analysis, GYRb1 was predicted as a virulent phage. The tRNA analysis revealed that the 2 tRNAs encoded in the GYRb1 genome correspond to arginine (Arg) and phenylalanine (Phe), respectively (Supplementary Table S2). Using CD-Search, 28 conserved domains were predicted in 20 ORFs (Supplementary Table S3). The genomic arrangement of these functional modules was visualized using the online tool Proksee Server (Figure 6). Furthermore, no recognizable antibiotic resistance genes or virulence factors were detected using CARD and VFDB.

### 3.6 Adsorption assay of phage GYRb1

**Figure 7:**
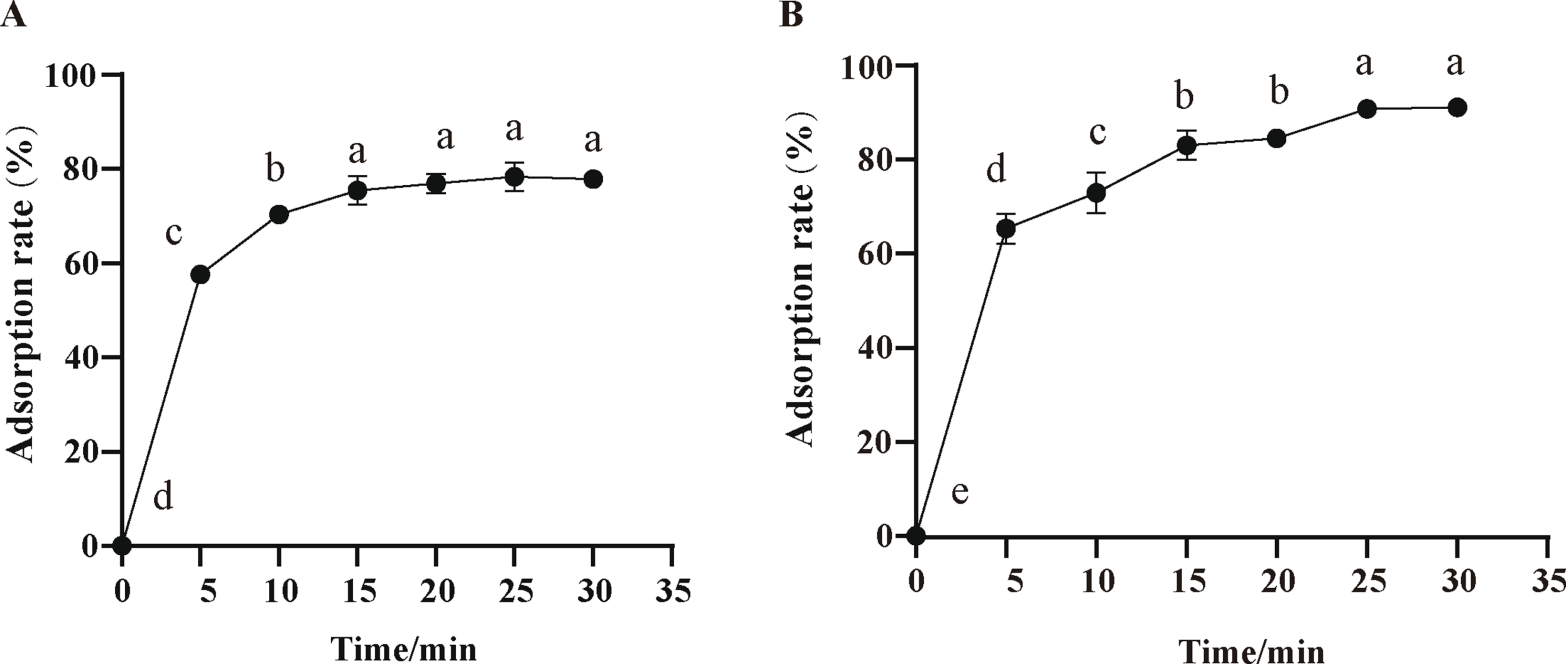
Adsorption kinetics of phage GYRb1. Adsorption curves on **(A)** *Xcc* 8004 and **(B)** *Xcc* 8004R. Phage GYRb1 was mixed with *Xcc* 8004 **(A)** or *Xcc* 8004R **(B)** at *MOI* = 0.1 and incubated at 28 °C with shaking. Samples were taken every 5 min for 30 min, centrifuged (12,000 × *g*), filtered (0.22 μm), and titers were determined at each time point. Data represent mean ± *SD* of three replicates. The adsorption rates of GYRb1 in *Xcc* 8004 or *Xcc* 8004R at each time point were compared by two way ANOVA method, and different letters indicate statistically significant differences (*p* < 0.05).

The adsorption rate of GYRb1 was higher on *Xcc* 8004R than on *Xcc* 8004 after mixing 30 min (*p <* 0.05). For *Xcc* 8004, adsorption plateaued 15 min post-infection, reaching 77.87% by 30 min. In contrast, adsorption to *Xcc* 8004R increased continuously, attaining 83.07% at 15 min, peaking at 25 min, and reaching to 91.08% at 30 min (Figure 7).

### 3.7 Phylogenetic analysis of phage GYRb1

**Figure 8:**
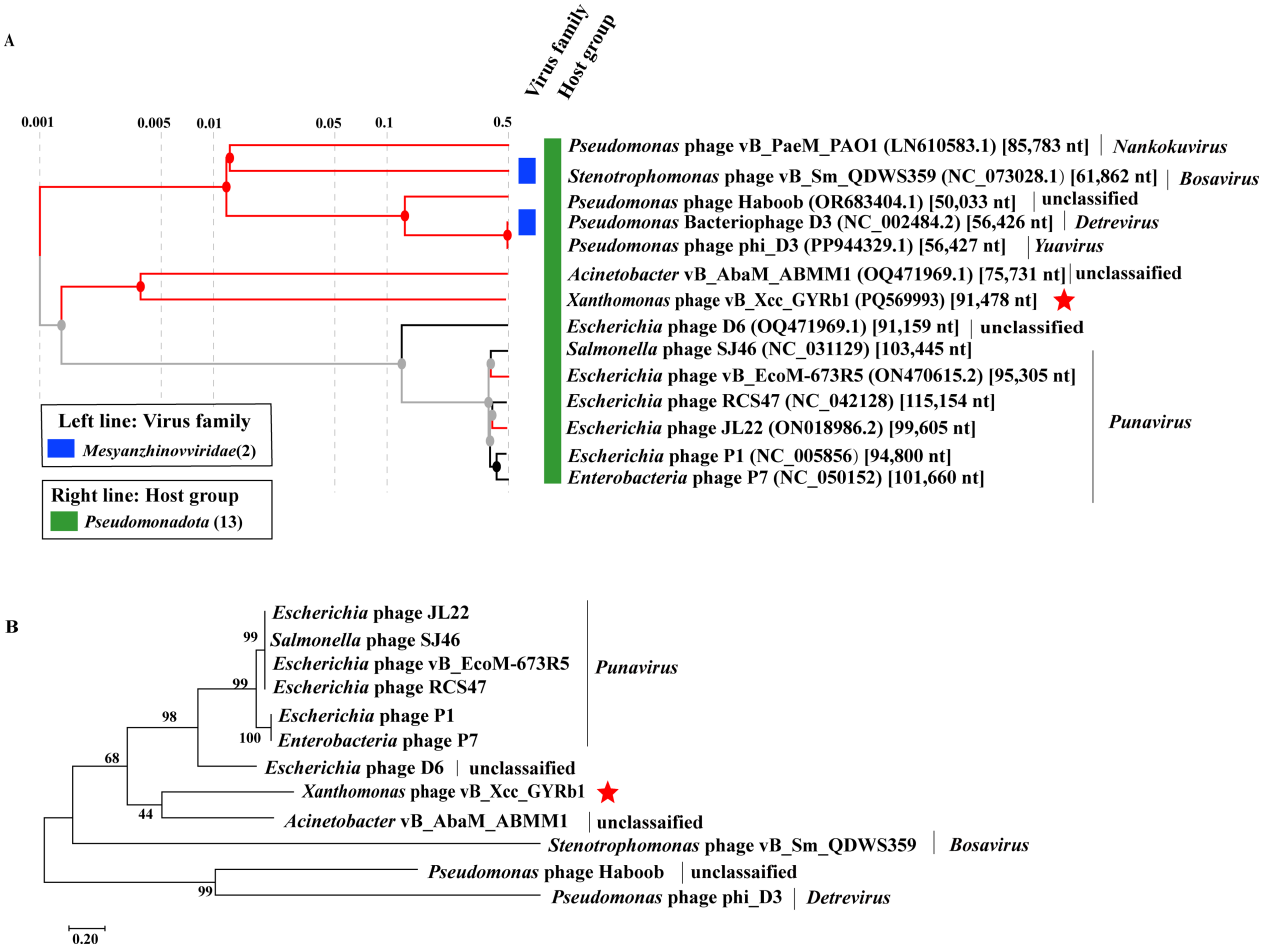
Phylogenetic analysis of phage GYRb1. **(A)** Viral proteomic tree of GYRb1 and its related phages. A combined dataset containing 14 phage genomes was used, with 8 additional related phage genomes retrieved from GenBank manually added (red branches) to the Viptree database. Branch lengths are shown on a logarithmic scale from the root of the tree. The inner nodes of the tree are shown as filled circles, each links to a genomic alignment of the sequences included in its subtree. The left panel indicated the virus family, and the right panel indicated the host group. **(B)** Phylogenetic tree of GYRb1 and its related phages by using MEGA (v11.0.13) based on the amino acid sequences of TerL. A total of 12 phages were included in the phylogenetic tree. *Pseudomonas* phage D3 and vB_PaeM_PAO1 were excluded due to the absence of the *TerL* gene. The evolutionary tree was constructed using the maximum likelihood method based on the AGA model with Gamma distribution, and 1,000 bootstrap replicates were performed to assess branch reliability. The red star represents phage GYRb1 isolated in this study.

A proteomic tree was constructed using the Viptree tool (Figure 8A). The results showed that GYRb1 clustered most closely with *Acinetobacter* phage vB_AbaM_ABMM1 (GenBank: OQ471969, abbreviated as ABMM1), which is an unclassified member of the class *Caudoviricetes.* Subsequently, this clade clustered with unclassified *Escherichia* phage D6 and six phages belonging to the *Punavirus* genus, including four *Escherichia* phages (vB_EcoM-673R, RCS47, JL22 and P1), *Salmonella* phage SJ46, and *Enterobacteria* phage P7.

A phylogenetic tree was constructed based on the amino acid sequence of TerL using MEGA software, and its topological structure was consistent with that of the proteomic tree (Figure 8B). Specifically, GYRb1 clustered with *Acinetobacter* phage ABMM1, followed by clustering with *Escherichia* phage D6 and the phages of *Punavirus* genus. All these phages were classified under the class *Caudoviricetes*.

### 3.8 Comparative genomic analysis of phage vB_Xcc_GYRb

**Figure 9:**
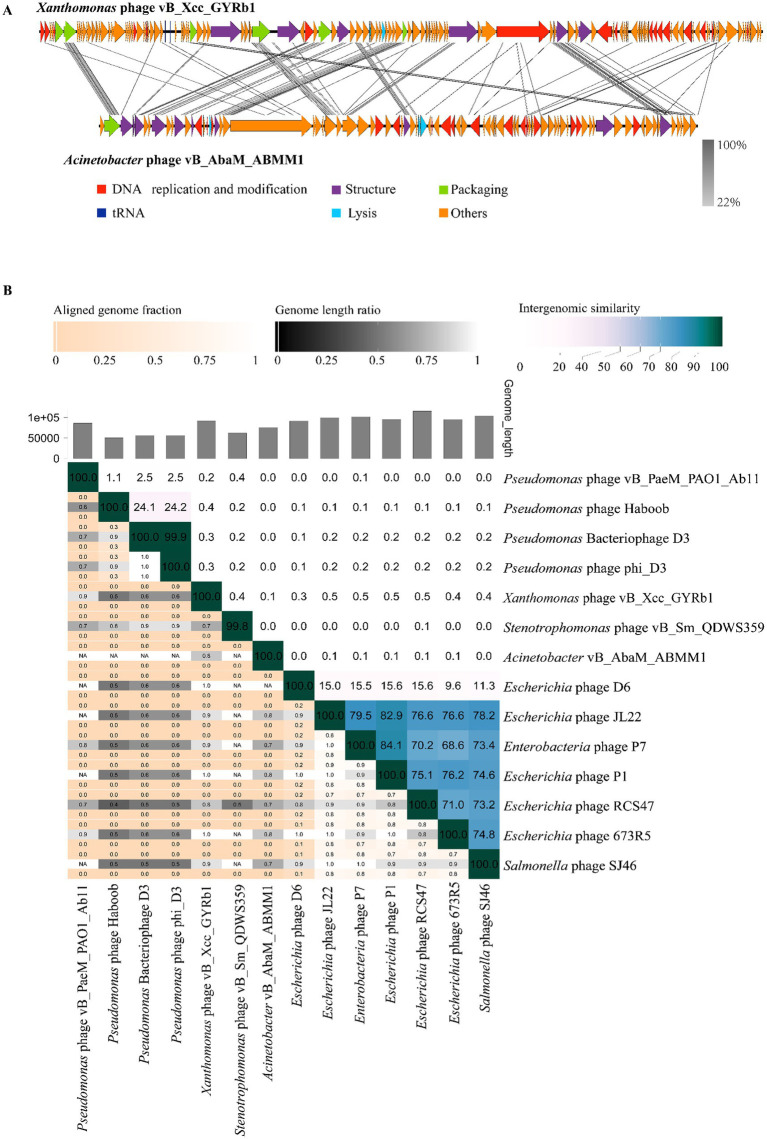
Comparative genomic analysis of GYRb1. **(A)** Genome sequence similarity between phage GYRb1 and ABMM1 was visualized using Easyfig software. Arrows indicate predicted ORFs, colored according to their predicted function. Gray lines connecting arrows indicate sequence similarity between the two phages, with color intensity indicating nucleotide sequence identity level. **(B)** Intergenomic similarity analysis of GYRb1 and the 13 most closely related phages calculated using VIRIDIC. The right half of this heatmap shows the similarity values between phage genomes. The left half of this heatmap shows the aligned genome fraction and genome length ratio. Phages sharing more than 95 and 70% intergenomic similarity are generally considered members of the same species and genus, respectively.

Although phylogenetic tree clustered GYRb1 and *Acinetobacter* phage ABMM1 into the same clade, BLASTn analysis revealed no significant sequence similarity between their whole-genome sequences. Further analysis using Easyfig software confirmed significant differences in the complete genome sequences of phage GYRb1 and ABMM1. Among the 128 proteins encoded by GYRb1, 63 (49.22%) shared homology with proteins encoded by ABMM1, with sequence similarity ranging from 22 to 76% (Figure 9A). The region exhibiting the highest similarity corresponds to ORF50, which is predicted to encode a nucleoid-associated transcriptional regulator of the MvaT/P16 family.

VIRIDIC analysis revealed that the intergenomic similarity values between phage GYRb1 and the selected phylogenetically related phages were all below 1%, with a maximum value of 0.5% (Figure 9B). These values are far below commonly used intergenomic similarity thresholds applied in phage taxonomy (Turner et al., 2021), supporting the conclusion that GYRb1 is highly divergent from the selected related phages.

**Figure 10:**
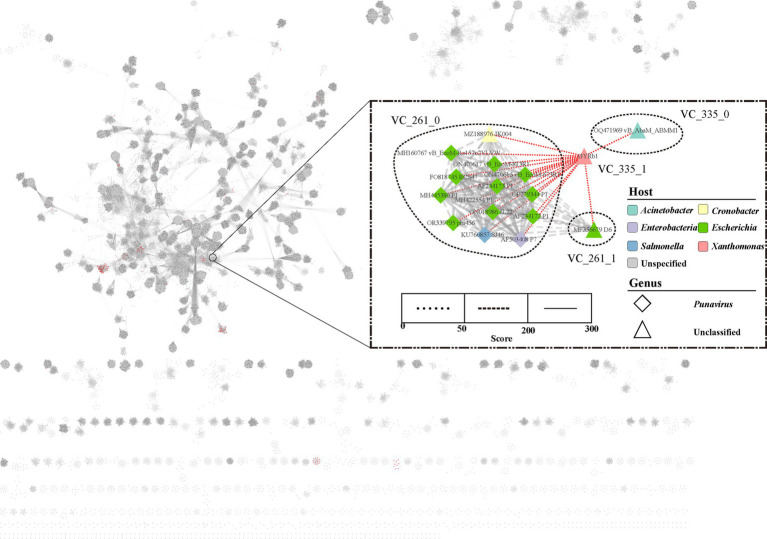
Genome network analysis. The interaction network was constructed using vConTACT2 and visualized with Cytoscape software, with reference to the INPHARED database (as of February 2025). Each circle (node) represents a viral genome, and connecting lines (edges) indicate genomic similarity between based on a significant number of shared protein clusters. Dashed lines delineate viral cluster (VCs). Different shapes (diamonds and triangles) represent viruses from different genera (taxonomic classification based on ICTV), and different colors distinguish the different hosts of the phage.

Genomic network analysis based on shared proteins revealed clustering of 26,278 phages, resulting in a total of 1,260,366 connections (Figure 10). Phage GYRb1 formed a singleton cluster (VC_335_1) and exhibited weak connections (scores below 50) with 15 phages distributed across clusters VC_261_0, VC_261_1, and VC_335_0. Cluster VC_261_0 included 12 phages of the *Punavirus* genus and one unclassified genus phage (*Cronobacter* phage JK004), while cluster VC_335_0 solely comprised *Acinetobacter* phage ABMM1. The host range of these phages included *Acinetobacter*, *Enterobacteria*, *Salmonella,* and *Cronobacter.*

### 3.9 *In vivo* antibacterial activity of phage GYRb1 against *Xcc* 8004 and *Xcc* 8004R

**Figure 11:**
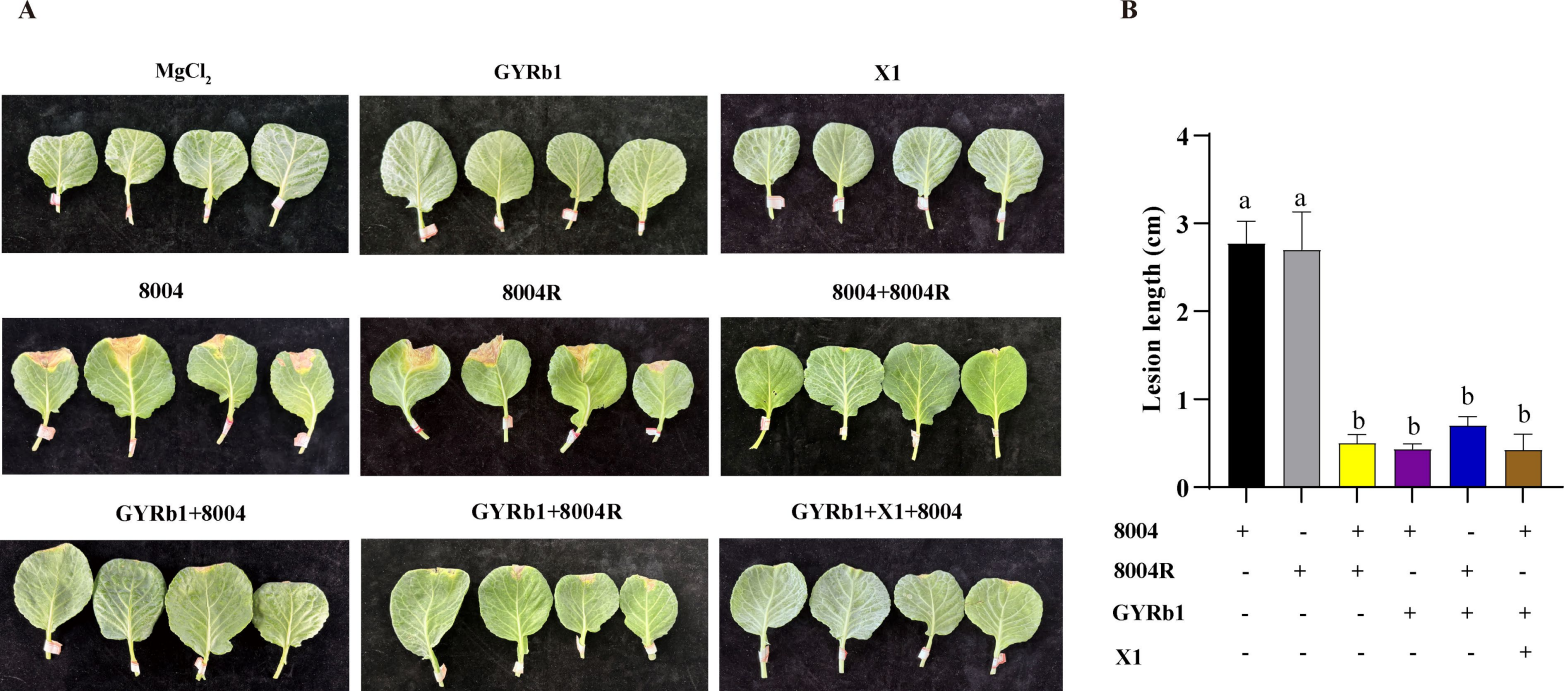
*In vivo* prophylactic inhibitory effect of phages against *Xcc*. **(A)** Leaf lesions observation after inoculation with *Xcc* strains. **(B)** Statistical analysis of V-shaped lesion lengths in each treatment group. For phage pretreatment, foliar spraying (FS) method was applied by selecting four fully expanded and similarly size leaves per plant and spraying them with 1 mL 10^6^ PFU/mL of phage suspension. After 24 h, bacterial cultures (OD_600_ = 0.1 in 10 mM MgCl_2_) were inoculated using the sterile clip method. Plants were incubated at 28 °C, 90% relative humidity with a 16 h photoperiod. Leaf lesion length was observed and measured 14 days after inoculation with *Xcc* strains. Each treatment group included six cabbage plants and three biological replicates were performed. MgCl_2_: 10 mM magnesium chloride solution (blank control). “+”: Phage was sprayed on the leaves using the FS method 24 h prior to bacterial inoculation. “−”: No phage treatment. Data are presented as mean ± SD of three independent replicates. Error bars represent SD. Different letters indicated significant differences among the treatment groups (*p* < 0.05).

The prophylactic inhibitory efficacy of phage GYRb1 against both wild-type *Xcc* 8004 and the phage-resistant strain *Xcc* 8004R was further evaluated using the FS method. After 14 days of treatment, no disease symptoms were observed on the leaves treated with MgCl_2_ buffer (blank control), GYRb1, or X1 (negative control) (Figure 11A). In contrast, inoculation of *Xcc* 8004 or *Xcc* 8004R (positive control) using the leaf-clipping resulted in typical severe V-shaped lesions (Figure 11A), and the pathogenicity of *Xcc* 8004R was comparable to that of *Xcc* 8004 (*p* > 0.05).

Pre-treatment with phage GYRb1 (Sprayed 24 h prior to bacterial inoculation) alleviated the severity of leaf symptoms (Figure 11A). Compared with the positive control group, GYRb1 pre-treatment reduced the lesion length caused by *Xcc* 8004 and *Xcc* 8004R by 84.04% (*p* < 0.01) and 74.07% (*p* < 0.01), respectively (Figure 11B). These results demonstrate that GYRb1 reduced disease severity caused by both strains under the tested *in vivo* conditions.

Spraying the phage cocktail (GYRb1 + X1) 24 h prior to *Xcc* 8004 inoculation effectively suppressed the development of leaf lesions (Figure 11A). The length of the V-shaped lesions was significantly reduced by 84.40% (*p <* 0.01), which was comparable to the lesion reduction rate achieved by GYRb1 pre-treatment (84.04%) (Figure 11B, *p* > 0.05). These findings indicate that the phage cocktail prophylactic treatment reduced disease severity caused by *Xcc* 8004, and no obvious antagonistic effect between the two phages was observed.

Notably, plants inoculated with a mixture of *Xcc* 8004 and *Xcc* 8004R developed less severe symptoms than plants inoculated with either strain alone (*p <* 0.01) (Figures 11A,B). This observation suggests possible inter-strain interactions during plant infection, but the mechanism remains unknown.

## 4 Discussion

Phages have demonstrated potential as biological control agents against plant bacterial diseases, but rapidly bacterial resistance can limit their long-term efficacy (Barron-Montenegro et al., 2022). In this study, we isolated a phage-resistant derivative of *Xcc* 8004 and used it as a selective host to isolate GYRb1, a phage that infects both the wild-type and the resistant mutant. The results support a practical proof-of-concept: phage-resistant mutants can serve as useful hosts for recovering additional candidate phages. However, mechanistic interpretations should be considered hypotheses supported by phenotypic and genomic associations rather than experimentally confirmed mechanisms. This study expands the available phage resource for addressing the phage-resistant pathogens and laid the foundation for further research on the interaction and co-evolution of bacteria and phages.

### 4.1 The mutant strain *Xcc* 8004R is resistant to phage X1

The phage resistant mutant *Xcc* 8004R was obtained after co-culture of *Xcc* 8004 with phage X1 and was selected. Phage-bacteria interactions often generate resistant variants through mutations affecting surface receptors, receptors masking, CRISPR-Cas systems, restriction-modification systems, abortive infection, or other defense pathways (Dy et al., 2014; Hampton et al., 2020; Bartnik et al., 2022; Egido et al., 2022). In *Xcc* 8004R, resequencing identified mutations in genes annotated as encoding an LPS core biosynthesis protein, PilY1 protein, N-acetylmuramoyl-L-alanine amidase, IS1404 transposase, and a conserved hypothetical protein. Because LPS and type IV pilus-associated proteins can participate in phage adsorption and host recognition, the LPS- and PilY1-associated mutations are plausible candidate contributors to the resistance phenotype (Marko et al., 2018; Warring et al., 2022; Torres-Quintero et al., 2024). However, these variants provide candidate resistance-associated loci but cannot be assigned causal roles without genetic validation, and the high residual adsorption of X1 to *Xcc* 8004R suggests that post-adsorption resistance mechanisms may also be involved.

The adsorption efficiency of X1 was lower on *Xcc* 8004R than on *Xcc* 8004 (Figure 1D), and *Xcc* 8004R also showed altered swimming and swarming motility (Figures 1E,F). These observations are consistent with changes in cell-surface architecture. Nevertheless, X1 still showed substantial adsorption to *Xcc* 8004R, the residual adsorption level remained high, suggesting that resistance may not be explained solely by impaired adsorption. Post-adsorption steps, including DNA injection, intracellular replication, host defense systems, or abortive infection, may also contribute to the resistant phenotype. Accordingly, the identified mutations should be described as candidate resistance-associated mutations rather than confirmed determinants of X1 resistance.

The growth of *Xcc* 8004R was similar to that of *Xcc* 8004, and the mutant retained pathogenicity in cabbage leaves (Figures 1C, 5, 11A). This finding is consistent with reports that phage resistance does not always impose a strong fitness or virulence cost and may depend on bacterial genotype, phage type, and environmental context (Castledine et al., 2022; Liang et al., 2025).

In the mixed infection assay, by *Xcc* 8004 and the phage resistant *Xcc* 8004R in planta caused disease symptoms were less severe than those caused by either strain alone (Figures 11A,B). This result suggests possible interactions between *Xcc* 8004 and *Xcc* 8004R during plant infection, but it should be interpreted cautiously because mixed-infection outcomes may depend on inoculum ratio, strain competition, niche occupation, and plant immune responses (Liyanapathiranage et al., 2022; Sadhukhan et al., 2024).

### 4.2 Biological characteristics of phage GYRb1

Using *Xcc* 8004R as the isolation host, we obtained phage GYRb1 from agricultural soil. This result supports the feasibility of using a resistant mutant as a selective host to enrich phages that remain active against resistant bacterial populations (Li et al., 2022). The ability of GYRb1 to infect both *Xcc* 8004 and *Xcc* 8004R, together with the different adsorption kinetics (Figures 5, 7), is consistent with the possibility that GYRb1 and X1 differ in host interaction mechanism. However, receptor use was not directly tested. Adsorption competition, receptor knockout or complementation, resistant mutant sequencing, or biochemical receptor binding assays would be required to confirm this hypothesis.

Genome annotation identified several GYRb1 proteins predicted to be associated with tail structure or host interaction, including a tail fiber domain-containing protein (ORF43), baseplate wedge protein (ORF85), tail-collar fiber protein (ORF87), minor tail protein (ORF88), and baseplate protein (ORF67). These proteins are plausible candidates for roles in adsorption or host recognition (Bertozzi Silva et al., 2016; Nobrega et al., 2018; Yehl et al., 2019; Li et al., 2022; Taslem Mourosi et al., 2022). However, annotation-based prediction is not sufficient to assign receptor-binding function. Comparative analysis showed that only the tail fiber proteins had detectable similarity, whereas other tail-associated proteins were highly divergent (Supplementary Table S4).

### 4.3 Genomic characteristics of GYRb1

The GYRb1 genome is circular dsDNA genome encoding 128 predicted ORFs, 36 showed no significant similarity to annotated proteins in the databases used and were distributed across four modules (Figure 6). The structural protein module includes a head protein (ORF79) and six tail-associated proteins that may participate in phage assembly and host interaction (Nobrega et al., 2018; Taslem Mourosi et al., 2022). The DNA packaging module contains the terminase small subunit (TerS, ORF7) and large subunit (TerL, ORF6), which are expected to mediate genome packaging in tailed phages. Terminase undertakes the enzymatic activity necessary for DNA packaging. TerS functions as a DNA recognition factor, binding to the packaging initiation site and transferring the DNA to the packaging motor of TerL (Lokareddy et al., 2022). Head assembly in dsDNA phages occurs in two stages. Capsid proteins assemble around a scaffold to form a precursor, which then undergoes a maturation process involving expansion to form the final mature head (Cheng et al., 2004). The prohead core protein protease (ORF39) cleaves the scaffold protein during precursor maturation. The capsid assembly protein (ORF47) and major capsid protein (ORF52) form an internal protein scaffold with other capsid subunits, playing crucial roles in capsid assembly (Davis et al., 2022). ORF68 encodes the defense against restriction A (DarA) protein, a component of the Dar system that which regulates the head size and the morphology of the capsid (Piya et al., 2017).

In the DNA replication and modification module, ORF109 encodes an exonuclease, ORF110 encodes a single-strand annealing protein, and both ORF30 and ORF104 encode DNA-binding proteins, all of which function in DNA replication, DNA packaging, and DNA repair. Holliday junction resolvase, encoded by ORF1 and ORF105, are specific endonucleases that cleave the linkage between two DNA strands to resolve holliday junctions (Gu et al., 2024). ORF90, ORF106 and ORF110 encode recombinase family proteins, which are enzymes that facilitate precise rearrangement of DNA sequences. Zinc-finger-containing proteins encoded by ORF25 and ORF124 harbor one of the most prevalent DNA-binding motifs in eukaryotes, participating in processes including RNA packaging, transcriptional activation, apoptosis regulation, protein folding and assembly, and lipid binding (Laity et al., 2001). ORF5, ORF50 and ORF92 encode transcription factors that regulate transcription. Helix-turn-helix (HTH) domain proteins, encoded by ORF2 and ORF95, are present in nearly all prokaryotes and some eukaryotes. In addition to transcription regulation, HTH domain are involved in DNA repair and replication, RNA metabolism, and protein–protein interactions under different signaling environments (Aravind et al., 2005). ORF35 encodes thioredoxin (TRX), a redox protein that acts as a cofactor in numerous cellular redox reactions.

In the lysis module includes endolysin (ORF59) and the outer membrane spanin component (o-spanin, ORF63), which are consistent with phage-induced host cell lysis (Kongari et al., 2018; Yehl et al., 2019; Skorynina et al., 2020).

The absence of recognizable antibiotic-resistance and bacterial virulence genes in the GYRb1 genome is encouraging, but it does not constitute a complete safety assessment. The annoation of CI/CII-like regulatory proteins raises the possibility of temperate behavior (Oppenheim et al., 2005; Fortier and Sekulovic, 2013), although through the use of PhageAI analysis, GYRb1 was predicted as a virulent phage. Therefore, lysogen formation assays, mitomycin C or UV induction tests, prophage screening of surviving colonies, and transduction risk assessment should be performed before GYRb1 is considered for field scale biocontrol development.

### 4.4 Phage GYRb1 may represent a divergent unclassified lineage within the class *Caudoviricetes*

Phylogenomic, proteomic, VIRIDIC, and vConTACT2 analyses indicate that GYRb1 is highly divergent from the related phages included in this study. GYRb1 clustered most closely with *Acinetobacter* phage ABMM1 in proteomic and TerL based analyses, but whole genome nucleotide similarity was extremely low, and GYRb1 formed a singleton viral cluster in the gene sharing network. These results support the conclusion that GYRb1 is taxonomically distinct from currently sampled related phages. GYRb1 may represent a previously unclassified genus level lineage within the class *Caudoviricetes*.

### 4.5 Prophylactic potential of GYRb1 against *Xcc* infections in plants

Phage GYRb1 inhibited the growth of *Xcc* 8004 and *Xcc* 8004R *in vitro* and prophylactic reduced lesion development in cabbage leaves (Figures 5, 11). These results indicate that GYRb1 has candidate biocontrol potential against both the parental strain and the X1-resistant mutant. Similar observations have been reported for phages isolated using resistant bacterial strains, which can retain activity against both wild-type and mutant hosts (Chen et al., 2024; Yoo et al., 2024). This therefore support the practical value of resistant mutant based phage isolation as a resource expansion strategy.

The GYRb1 + X1 cocktail reduced OD_600_-based regrowth in vitro compared with some single-phage treatments and reduced disease severity in cabbage leaves under the tested prophylactic conditions. However, because resistant colonies were not isolated and genotyped after treatment, these data do not directly demonstrate suppression of emergence of resistant populations. Reduced regrowth that is consistent with a partial delay in resistance emergence.

Environmental stability under field conditions represents critical factor restricting the practical application of GYRb1. UV exposure assay revealed that UV irradiation caused a time-dependent reduction in GYRb1 titer. Accordingly, reasonable management strategies are recommended for field foliar application. Future studies should evaluate the UV stability of GYRb1 under natural field conditions to further inform these protective strategies.

A further limitation is the possible temperate nature of GYRb1. Temperate phages can lysogenize bacterial hosts, reduce immediate killing, and potentially contribute to horizontal gene transfer (Brüssow et al., 2004; Feiner et al., 2015). Although GYRb1 was considered as a virulent phage based on a machine learning PhageAI analysis, and showed bacterial suppression and disease reduction, the presence of CI/CII-like gene warrants caution. If GYRb1 proves temperate, genetic modification to remove lysogeny associated functions may be required before it can be developed as a safer biocontrol agent (Sykilinda et al., 2018; Chang et al., 2019).

Overall, this study provides proof of concept evidence that phage-resistant mutants can be used as selective hosts to obtain additional phages with activity against resistant bacterial populations. The strategy may help expand phage resources for agricultural disease control, but its generality and mechanistic basis require validation using multiple independently derived resistant mutants, diverse field isolates, and direct receptor identification assays.

## 5 Conclusion

In this study, we isolated a spontaneous X1 resistant derivative of *Xcc* 8004, designated *Xcc* 8004R. Compared with the parental strain *Xcc* 8004, *Xcc* 8004R exhibited altered colony morphology, altered motility, reduced but still substantial X1 adsorption, and retained pathogenicity in cabbage leaves. Whole genome resequencing identified mutations in genes annotated as encoding LPS core biosynthesis proteins, PilY1 N-acetylmuramoyl-L-alanine amidase, IS1404 transposase, and conserved hypothetical proteins. These loci are candidate resistance associated mutations, but their causal roles require further validation. Using *Xcc* 8004R as a selective host, we isolated phage GYRb1 from cabbage field soil. GYRb1 infected both *Xcc* 8004 and *Xcc* 8004R, suppressed bacterial growth in vitro, and reduced lesion development in a controlled prophylactic cabbage assay. Genomic and phylogenomic analyses showed that GYRb1 has a 91,478-bp dsDNA genome encoding 128 predicted ORFs and two tRNAs and is highly divergent from currently available related phages, suggesting that it represents a candidate unclassified lineage within *Caudoviricetes*. No recognizable antibiotic resistance or bacterial virulence genes were detected; however, CI/CII-like regulatory proteins warrant further investigation of lysogeny and safety. Overall, this work provides proof of concept evidence that phage-resistant mutants can be used as selective hosts to expand candidate phage resources for *Xcc* biocontrol, while highlighting the need for receptor identification, resistance frequency analysis, broader host range testing, lysogeny assessment, and field relevant efficacy studies.

## Data Availability

The data presented in this study can be found in the NCBI (https://www.ncbi.nlm.nih.gov/genbank), under accession PQ569993.

## Footnotes

1
http://topaz.gatech.edu/GeneMark/genemarks.cgi

2
http://rast.nmpdr.org/rast.cgi

3
https://blast.ncbi.nlm.nih.gov/Blast.cgi

4
https://lowelab.ucsc.edu/tRNAscan-SE/

5
https://www.Ncbi.nlm.Nih.gov/Structure/cdd/wrpsb.cgi

6
https://proksee.ca/

7
https://app.phage.ai

8
https://www.genome.jp/viptree

9
http://rhea.icbm.unioldenburg.de/viridic/
