# Supplementary material for: Isolation of a complementary bacteriophage from a phage-resistant mutant expands control strategies against Xanthomonas campestris
Source: Front Microbiol. 2026 Aug 3;17:1853113. doi: 10.3389/fmicb.2026.1853113 (PMC13478112; doi:10.3389/fmicb.2026.1853113)
Supplement: Supplementary file 1 [file Data_Sheet_1.DOCX]

**Supplementary Table S1.** Functional assignments of putative ORFs in the phage GYRb1 genome.

| **Group** | **ORF** | **Start** | **Stop** | **strand** | **Predicted function** | **E-value** | **Identity (%)** | **Accession** |
| --- | --- | --- | --- | --- | --- | --- | --- | --- |
| Structure | 43 | 21719 | 25534 | + | tail fiber domain-containing protein | 7.00E-54 | 50.00 | WJJ55131.1 |
|  | 54 | 37655 | 39298 | + | tail connector protein | 0 | 59.20 | DAY72174.1 |
|  | 67 | 45955 | 46551 | + | baseplate protein | 1.00E-56 | 64.66 | DAY72182.1 |
|  | 79 | 51801 | 55466 | + | Internal head protein | 5.00E-82 | 56.05 | DAY72102.1 |
|  | 85 | 65361 | 66854 | + | baseplate wedge protein | 0 | 56.14 | DAY72193.1 |
|  | 87 | 67816 | 68193 | + | tail-collar fiber protein | 1.00E-26 | 47.24 | DAY72196.1 |
|  | 88 | 68205 | 69827 | + | minor tail protein | 6.00E-23 | 26.21 | DAM58653.1 |
| Packaging | 6 | 2060 | 3145 | + | Terminase small subunit | 6.00E-38 | 33.00 | DAY72098.1 |
|  | 7 | 3138 | 4709 | + | large terminase | 0 | 65.70 | DAY72099.1 |
|  | 39 | 19095 | 19952 | + | Prohead core protein protease | 6.00E-60 | 49.77 | DAV81068.1 |
|  | 47 | 26942 | 29224 | + | capsid assembly protein | 0 | 60.97 | DAF90844.1 |
|  | 52 | 35314 | 36966 | + | Major capsid protein | 0 | 57.74 | DAV45181.1 |
|  | 68 | 46620 | 47102 | + | Defence against restriction A N-terminal | 1.00E-51 | 51.28 | DAY72187.1 |
| DNA replication and modification | 1 | 192 | 686 | + | holliday junction resolvase | 2.00E-38 | 51.85 | DAG84892.1 |
|  | 2 | 729 | 1343 | + | helix-turn-helix domain protein | 2.00E-47 | 47.46 | DAY72090.1 |
|  | 5 | 1811 | 2041 | + | Transcription initiation factor IIE, alpha FINGER, Transcription | 1.00E-06 | 36.54 | DAL54615.1 |
|  | 25 | 11946 | 12320 | + | zinc-finger-containing protein | 3.00E-44 | 55.08 | WEM34393.1 |
|  | 30 | 13720 | 13929 | + | DNA-binding protein | 8.00E-09 | 53.85 | QWY14273.1 |
|  | 35 | 17975 | 18184 | + | thioredoxin | 5.00E-22 | 42.71 | CAH1087899.1 |
|  | 48 | 30122 | 32968 | + | baseplate hub subunit and tail lysozyme | 5.00E-14 | 30.56 | YP_010662706.1 |
|  | 50 | 33621 | 34700 | + | Transcriptional regulator MvaT, P16 subunit regulator, nucleoid associated protein.3A | 1.00E-92 | 48.20 | DAY72169.1 |
|  | 81 | 55952 | 57865 | + | Metallo-peptidase family M12B Reprolysin-like | 3.00E-22 | 35.53 | DAY17008.1 |
|  | 82 | 57866 | 64582 | + | Chromatin remodeling complex ATPase | 0 | 42.99 | DAY72108.1 |
|  | 90 | 70495 | 72393 | - | serine recombinase | 0 | 55.36 | ULR75073.1 |
|  | 92 | 72730 | 73098 | - | transcriptional regulator | 3.00E-04 | 35.21 | UXY92546.1 |
|  | 95 | 74050 | 74295 | - | Helix-turn-helix XRE-family like protein | 3.00E-04 | 37.50 | DAJ54340.1 |
|  | 103 | 77052 | 77519 | - | phosphoheptose isomerase | 1.00E-30 | 47.86 | YP_009594920.1 |
|  | 104 | 77601 | 78116 | - | ssDNA-binding protein | 1.00E-60 | 59.22 | QZI85846.1 |
|  | 105 | 78116 | 78763 | - | RusA-like Holliday junction resolvase | 9.00E-10 | 31.09 | YP_008130294.1 |
|  | 106 | 78766 | 79725 | - | DNA recombination-dependent growth factor C | 4.00E-93 | 45.60 | XAI97162.1 |
|  | 109 | 80811 | 81437 | - | exonuclease | 2.00E-45 | 45.37 | QYW06368.1 |
|  | 110 | 81443 | 82474 | - | single strand annealing protein | 6.00E-69 | 49.59 | CAJ1864403.1 |
|  | 113 | 83540 | 84217 | - | Repressor protein CI | 7.00E-22 | 31.53 | DAP51150.1 |
|  | 116 | 85030 | 85293 | + | CII protein | 3.00E-04 | 58.54 | DAN94605.1 |
|  | 124 | 89475 | 89684 | + | TraR/DksA C4-type zinc finger protein | 0.004 | 37.14 | WPJ21818.1 |
| Lysis | 63 | 43788 | 44093 | + | outer membrane spanin component | 5.00E-18 | 48.19 | YP_009851393.1 |
|  | 59 | 41916 | 42578 | + | endolysin | 3.00E-63 | 52.79 | YP_010106983.1 |
| other protein | 3 | 1340 | 1585 | + | no hits |  |  |  |
|  | 4 | 1589 | 1807 | + | no hits |  |  |  |
|  | 8 | 4806 | 5048 | + | no hits |  |  |  |
|  | 9 | 5045 | 5284 | + | hypothetical protein DLP3_068 | 3.00E-06 | 47.83% | QIN95231.1 |
|  | 10 | 5281 | 5538 | + | hypothetical protein | 0.002 | 44.68% | WJJ54969.1 |
|  | 11 | 5528 | 5947 | + | no hits |  |  |  |
|  | 12 | 5970 | 6152 | + | no hits |  |  |  |
|  | 13 | 6145 | 6702 | + | hypothetical protein R2B_p033 | 1.00E-11 | 39.20% | ASN73070.1 |
|  | 14 | 6666 | 7205 | + | hypothetical protein AVU38_gp197 | 1.00E-08 | 47.06% | YP_009213046.1 |
|  | 15 | 7196 | 7573 | + | no hits |  |  |  |
|  | 16 | 7557 | 7820 | + | no hits |  |  |  |
|  | 17 | 7814 | 8767 | + | hypothetical protein | 6.00E-26 | 48.91% | URC09300.1 |
|  | 18 | 8764 | 9063 | + | no hits |  |  |  |
|  | 19 | 9127 | 9369 | + | hypothetical protein F394_gp10 | 3.00E-10 | 40.00% | YP_007007699.1 |
|  | 20 | 9366 | 10928 | + | hypothetical protein DLP3_125 | 5.00E-26 | 83.61% | QIN95288.1 |
|  | 21 | 10918 | 11151 | + | hypothetical protein Acf1_00015 | 1.00E-16 | 58.21% | QXV72212.1 |
|  | 22 | 11144 | 11386 | + | no hits |  |  |  |
|  | 23 | 11383 | 11706 | + | no hits |  |  |  |
|  | 24 | 11699 | 11953 | + | no hits |  |  |  |
|  | 26 | 12317 | 13204 | + | hypothetical protein KS10_gp24 | 8.00E-14 | 76.19% | YP_002221440.1 |
|  | 27 | 13183 | 13425 | + | no hits |  |  |  |
|  | 28 | 13425 | 13526 | + | no hits |  |  |  |
|  | 29 | 13523 | 13723 | + | hypothetical protein PP740_gp034 | 1.00E-06 | 44.64% | YP_010657481.1 |
|  | 31 | 14047 | 14511 | + | hypothetical protein UFOVP467_13 | 3.00E-14 | 40.74% | CAB4144953.1 |
|  | 32 | 14546 | 15343 | + | hypothetical protein DLP3_124 | 7.00E-51 | 41.46% | QIN95287.1 |
|  | 33 | 15340 | 15675 | + | no hits |  |  |  |
|  | 34 | 17196 | 17390 | + | no hits |  |  |  |
|  | 36 | 18181 | 18609 | + | no hits |  |  |  |
|  | 37 | 18591 | 18812 | + | hypothetical protein Acj133p051 | 0.002 | 52.38% | YP_004300632.1 |
|  | 38 | 18809 | 19018 | + | no hits |  |  |  |
|  | 40 | 19956 | 20723 | + | hypothetical protein | 7.00E-70 | 46.98% | DAY72166.1 |
|  | 41 | 20720 | 21208 | + | hypothetical protein | 1.00E-46 | 51.70% | DAS32329.1 |
|  | 42 | 21216 | 21719 | + | hypothetical protein | 8.00E-32 | 55.45% | DAY72168.1 |
|  | 44 | 25547 | 25981 | + | hypothetical protein | 5.00E-07 | 34.95% | USV40964.1 |
|  | 45 | 25985 | 26485 | + | hypothetical protein | 9.00E-21 | 40.13% | USV40963.1 |
|  | 46 | 26565 | 26903 | + | hypothetical protein UFOVP47_68 | 5.00E-13 | 36.97% | CAB4241108.1 |
|  | 49 | 32975 | 33484 | - | no hits |  |  |  |
|  | 51 | 34705 | 35208 | + | hypothetical protein | 4.00E-55 | 50.30% | DAY72170.1 |
|  | 53 | 36983 | 37642 | + | hypothetical protein | 9.00E-08 | 40.95% | DAY72172.1 |
|  | 55 | 39335 | 40024 | + | hypothetical protein | 1.00E-79 | 55.97% | DAY72175.1 |
|  | 56 | 40094 | 40921 | + | hypothetical protein | 4.00E-78 | 48.06% | DAY72176.1 |
|  | 57 | 40944 | 41699 | + | hypothetical protein | 6.00E-68 | 45.77% | DAY72177.1 |
|  | 58 | 41755 | 41916 | + | hypothetical protein | 9.00E-05 | 36.54% | DAY72178.1 |
|  | 60 | 42575 | 42982 | + | no hits |  |  |  |
|  | 61 | 43001 | 43348 | + | no hits |  |  |  |
|  | 62 | 43348 | 43791 | + | Protein of unknown function (DUF2570) | 0.007 | 32.09% | DAM25262.1 |
|  | 64 | 44157 | 44657 | + | hypothetical protein | 3.00E-75 | 61.05% | DAY72179.1 |
|  | 65 | 44657 | 45040 | + | hypothetical protein | 7.00E-32 | 48.00% | DAY72180.1 |
|  | 66 | 45044 | 45940 | + | hypothetical protein | 5.00E-129 | 59.06% | DAY72181.1 |
|  | 69 | 47215 | 48204 | + | hypothetical protein Bhz59_00069 | 8.00E-26 | 37.57% | WVW77455.1 |
|  | 70 | 48276 | 48848 | + | no hits |  |  |  |
|  | 71 | 48909 | 49301 | + | no hits |  |  |  |
|  | 72 | 49303 | 49539 | + | hypothetical protein HWC11_gp016 | 2.00E-16 | 50.00% | YP_009844362.1 |
|  | 73 | 49632 | 49898 | + | hypothetical protein RCXUPER_210 | 1.00E-16 | 48.86% | UVD36628.1 |
|  | 74 | 49898 | 50191 | + | hypothetical protein Cygsa01_00120 | 3.00E-12 | 41.98% | XAI71166.1 |
|  | 75 | 50195 | 50566 | + | hypothetical protein | 8.00E-15 | 39.50% | UOL49211.1 |
|  | 76 | 50563 | 51225 | + | hypothetical protein CPT_Sonora_063 | 5.00E-16 | 54.10% | QYW02164.1 |
|  | 77 | 51222 | 51539 | + | no hits |  |  |  |
|  | 78 | 51536 | 51772 | + | no hits |  |  |  |
|  | 80 | 55479 | 55943 | + | no hits |  |  |  |
|  | 83 | 64590 | 65015 | + | hypothetical protein | 2.00E-14 | 33.33% | DAY17006.1 |
|  | 84 | 65072 | 65359 | + | hypothetical protein | 3.00E-27 | 54.35% | DAF90850.1 |
|  | 86 | 66858 | 67739 | + | hypothetical protein | 4.00E-76 | 44.67% | DAY72194.1 |
|  | 89 | 69827 | 70483 | + | hypothetical protein CH36_gp22 | 2.00E-13 | 32.22% | YP_009008377.1 |
|  | 91 | 72454 | 72660 | - | no hits |  |  |  |
|  | 93 | 73358 | 73780 | - | no hits |  |  |  |
|  | 94 | 73820 | 74053 | - | hypothetical protein JC1_2 | 7.00E-21 | 51.39% | ULR75074.1 |
|  | 96 | 74292 | 74456 | - | no hits |  |  |  |
|  | 97 | 74802 | 75626 | - | hypothetical protein | 8.00E-104 | 60.58% | DAE50494.1 |
|  | 98 | 75623 | 75934 | - | hypothetical protein QAY88_gp32 | 6.00E-09 | 66.67% | YP_010742786.1 |
|  | 99 | 75931 | 76215 | - | hypothetical protein KEM13_gp65 | 9.00E-30 | 63.41% | YP_010052623.1 |
|  | 100 | 76218 | 76553 | - | hypothetical protein | 6.00E-15 | 44.12% | DAG21468.1 |
|  | 101 | 76550 | 76753 | - | hypothetical protein | 4.00E-06 | 43.75% | WOZ57210.1 |
|  | 102 | 76732 | 77055 | - | DUF4031 domain-containing protein | 8.00E-40 | 69.07% | YP_009792777.1 |
|  | 107 | 79788 | 79958 | - | no hits |  |  |  |
|  | 108 | 79965 | 80807 | - | hypothetical protein vBSbQDWS_69 | 1.00E-20 | 37.65% | UEP18749.1 |
|  | 111 | 82557 | 82697 | - | hypothetical protein | 0.001 | 46.51% | QIQ62525.1 |
|  | 112 | 82858 | 83517 | - | no hits |  |  |  |
|  | 114 | 84404 | 84676 | + | hypothetical protein | 1.00E-09 | 43.42% | DAG78019.1 |
|  | 115 | 84673 | 84783 | + | no hits |  |  |  |
|  | 117 | 85290 | 85640 | + | hypothetical protein UFOVP259_20 | 4.00E-26 | 49.04% | CAB4132317.1 |
|  | 118 | 85637 | 85822 | + | no hits |  |  |  |
|  | 119 | 85910 | 86857 | + | Protein of unknown function DUF1376 | 4e-27 | 53.92% | CAB4126104.1 |
|  | 120 | 86854 | 88383 | + | hypothetical protein JK004_33 | 2.00E-103 | 42.95% | QVJ82390.1 |
|  | 121 | 88380 | 88964 | + | hypothetical protein vBPaeMUSP18_44 | 1.00E-33 | 37.50% | QLI49432.1 |
|  | 122 | 88961 | 89167 | + | no hits |  |  |  |
|  | 123 | 89240 | 89482 | + | no hits |  |  |  |
|  | 125 | 89681 | 89962 | + | no hits |  |  |  |
|  | 126 | 89959 | 90753 | + | hypothetical protein FDH89_gp52 | 3.00E-15 | 33.88% | YP_009604352.1 |
|  | 127 | 90758 | 91099 | + | hypothetical protein GOVbin132_11 | 5.00E-17 | 42.57% | QDP49867.1 |
|  | 128 | 91096 | 91317 | + | no hits |  |  |  |

**Supplementary Table S2.** The tRNAs predicted in the phage GYRb1 genome.

| tRNA | start | stop | Function |
| --- | --- | --- | --- |
| 1 | 15887 | 15966 | tRNA-Arg-GCG |
| 2 | 16768 | 16840 | tRNA-Phe-GAA |

**Supplementary Table S3.** Conserved domains found in ORFs of phage GYRb1 genome.

| ORF | From | To | Short name | E-Value | Bitscore | Accession |
| --- | --- | --- | --- | --- | --- | --- |
| 6 | 110 | 195 | XtmA | 1.35E-22 | 66.1297 | COG3728 |
| 25 | 46 | 76 | DUF6440 super family | 2.14E-04 | 50.202 | cl45417 |
| 35 | 8 | 104 | CnoX | 4.70E-39 | 125.702 | COG3118 |
| 43 | 115 | 1000 | FhaB superfamily | 7.33E-07 | 54.002 | cl27105 |
| 43 | 1159 | 1221 | Peptidase_S74 | 1.20E-05 | 43.7732 | pfam13884 |
| 43 | 1053 | 1087 | Shufflon_N superfamily | 0.00056749 | 43.4463 | cl20035 |
| 59 | 22 | 199 | Muramidase | 5.33E-67 | 203.643 | pfam11860 |
| 82 | 208 | 425 | YtxK superfamily | 1.56E-12 | 71.1337 | cl28092 |
| 82 | 868 | 1207 | HepA superfamily | 3.61E-10 | 65.2465 | cl33945 |
| 82 | 942 | 1504 | COG4646 superfamily | 1.31E-06 | 54.1012 | cl18793 |
| 82 | 1463 | 1616 | P-loop_NTPase superfamily | 1.70E-05 | 46.3157 | cl38936 |
| 88 | 305 | 382 | PTR superfamily | 6.02E-07 | 46.7755 | cl15092 |
| 88 | 175 | 252 | PTR superfamily | 1.09E-05 | 42.9235 | cl15092 |
| 88 | 24 | 422 | AidA superfamily | 6.64E-05 | 45.7075 | cl34620 |
| 90 | 44 | 194 | Ser_Recombinase | 8.20E-24 | 97.3313 | cd00338 |
| 90 | 41 | 448 | SpoIVCA | 7.47E-23 | 100.87 | COG1961 |
| 90 | 31 | 120 | HTH_ARSR superfamily | 1.64E-06 | 43.3515 | cl46857 |
| 95 | 3 | 65 | HipB | 2.79E-10 | 51.1508 | COG1396 |
| 99 | 6 | 86 | DUF6378 superfamily | 5.44E-06 | 40.658 | cl45293 |
| 103 | 3 | 83 | DUF4031 | 5.44E-26 | 91.9149 | pfam13223 |
| 104 | 1 | 164 | RPA_2b-aaRSs_OBF_like superfamily | 3.67E-74 | 219.811 | cl09930 |
| 106 | 1 | 312 | RdgC | 6.16E-120 | 346.755 | COG2974 |
| 110 | 24 | 214 | RecT superfamily | 4.05E-42 | 144.585 | cl04285 |
| 113 | 132 | 217 | COG2932 | 3.35E-18 | 77.3094 | COG2932 |
| 114 | 24 | 78 | YdaS | 4.79E-16 | 65.7031 | COG4197 |
| 119 | 10 | 127 | YdaU | 3.30E-38 | 131.657 | COG3756 |
| 120 | 27 | 461 | DnaB | 3.03E-168 | 481.884 | COG0305 |
| 127 | 9 | 102 | DUF968 superfamily | 4.94E-13 | 59.9808 | cl23554 |

**Supplementary Table S4.** Tail-associated proteins of phage GYRb1 and X1.

| **Functional category** | **GYRb1** | **X1** | **E-value** | **Identity (%)** |
| --- | --- | --- | --- | --- |
| Tail fiber protein | ORF43: tail fiber domain-containing protein | ORF281: tail fiber protein | 2e-47 | 47% |
| Minor tail protein | ORF88: minor tail protein | ORF247: minor tail protein | No significant similarity | |
| Tail tube-related protein | ORF54: tail connector protein | ORF1: tail tube associated baseplate protein | No significant similarity | |
|  | ORF87: tail-collar fiber protein | ORF49: tail tube protein |  |  |
| Baseplate components | ORF67: baseplate protein | ORFs (2, 35, 36, 37, 309): baseplate wedge protein | No significant similarity | |
|  | ORF85: baseplate wedge protein |  |  |  |
|  | ORF48: baseplate hub subunit and tail lysozyme | ORF103: baseplate hub protein |  |  |

**Supplementary Table S5.** Optimal MOI for phage infection of *Xcc* 8004 and *Xcc* 8004R.

| **Host bacterium (CFU/mL)** | **Phage (PFU/mL)** | **MOI** | ***Xcc* 8004 Titer (PFU/mL)** | ***Xcc* 8004R Titer (PFU/mL)** |
| --- | --- | --- | --- | --- |
| 10^8^ | 10^5^ | 0.001 | 2.87×10^4^ | 6.27×10^5^ |
| 10^6^ | 10^6^ | 0.1 | 1.69×10^5^ | 1.95×10^6^ |
| 10^5^ | 10^6^ | 1 | 2.95×10^4^ | 7.53×10^4^ |
| 10^4^ | 10^6^ | 10 | 3.10×10^3^ | 1.13×10^4^ |
| 10^3^ | 10^6^ | 100 | 4.33×10^2^ | 4.30×10^3^ |

**Supplementary Figure S1.** Sequence comparison of lipopolysaccharide core biosynthesis proteins between *Xcc* 8004 and *Xcc* 8004R

**Supplementary Figure S2.** Sequence comparison of N-acetylmuramyl-L-alanine amidase between *Xcc* 8004 and *Xcc* 8004R.

**Supplementary Figure S3.** Sequence comparison of IS1404 transposase between *Xcc* 8004 and *Xcc* 8004R.

**Supplementary Figure S4.** Sequence comparison of conserved putative proteins between *Xcc* 8004 and *Xcc* 8004R.

**Supplementary Figure S5.** Sequence comparison of PilY1 protein between *Xcc* 8004 and *Xcc* 8004R.
